# Duplication and concerted evolution of MiSp-encoding genes underlie the material properties of minor ampullate silks of cobweb weaving spiders

**DOI:** 10.1186/s12862-017-0927-x

**Published:** 2017-03-14

**Authors:** Jannelle M. Vienneau-Hathaway, Elizabeth R. Brassfield, Amanda Kelly Lane, Matthew A. Collin, Sandra M. Correa-Garhwal, Thomas H. Clarke, Evelyn E. Schwager, Jessica E. Garb, Cheryl Y. Hayashi, Nadia A. Ayoub

**Affiliations:** 1 Department of Biology, Washington and Lee University, Lexington, VA USA; 2 Department of Biology, University of California, Riverside, CA USA; 3 Department of Biological Sciences, University of Massachusetts, Lowell, MA USA

**Keywords:** Theridiidae, *Latrodectus*, *Steatoda*, Silk glands, Gene expression, Silk proteins, Spidroin gene family

## Abstract

**Background:**

Orb-web weaving spiders and their relatives use multiple types of task-specific silks. The majority of spider silk studies have focused on the ultra-tough dragline silk synthesized in major ampullate glands, but other silk types have impressive material properties. For instance, minor ampullate silks of orb-web weaving spiders are as tough as draglines, due to their higher extensibility despite lower strength. Differences in material properties between silk types result from differences in their component proteins, particularly members of the spidroin (spider fibroin) gene family. However, the extent to which variation in material properties within a single silk type can be explained by variation in spidroin sequences is unknown. Here, we compare the minor ampullate spidroins (MiSp) of orb-weavers and cobweb weavers. Orb-web weavers use minor ampullate silk to form the auxiliary spiral of the orb-web while cobweb weavers use it to wrap prey, suggesting that selection pressures on minor ampullate spidroins (MiSp) may differ between the two groups.

**Results:**

We report complete or nearly complete *MiSp* sequences from five cobweb weaving spider species and measure material properties of minor ampullate silks in a subset of these species. We also compare MiSp sequences and silk properties of our cobweb weavers to published data for orb-web weavers. We demonstrate that all our cobweb weavers possess multiple *MiSp* loci and that one locus is more highly expressed in at least two species. We also find that the proportion of β-spiral-forming amino acid motifs in MiSp positively correlates with minor ampullate silk extensibility across orb-web and cobweb weavers.

**Conclusions:**

MiSp sequences vary dramatically within and among spider species, and have likely been subject to multiple rounds of gene duplication and concerted evolution, which have contributed to the diverse material properties of minor ampullate silks. Our sequences also provide templates for recombinant silk proteins with tailored properties.

**Electronic supplementary material:**

The online version of this article (doi:10.1186/s12862-017-0927-x) contains supplementary material, which is available to authorized users.

## Background

**Fig. 1:**
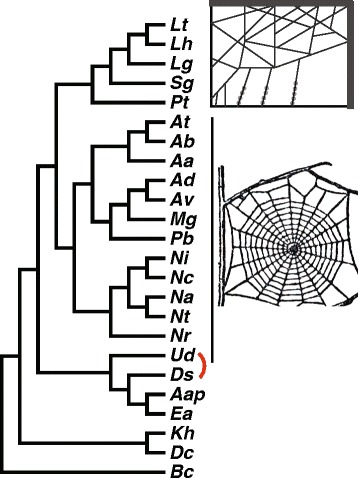
Relationships among spider species in this study. See Table 1 for species abbreviations and family status. We focused on comparing minor ampullate silks between species that build cobwebs, a disordered three-dimensional web that typically catches walking prey (top graphic) and species that build orb-webs, a two dimensional wagon-wheel shaped web that catches flying prey (bottom graphic, from http://cliparts.co/spiders-web-clip-art). Relationships are based on a compilation of published spider phylogenies (see Methods). The *red* elbow indicates that one study [38] found the relationship shown in the figure, while another [39] switched the placement of the families to which these species belong

Orb-web and cobweb weaving spiders in the superfamily Araneoidea use silk fibers and glues for a variety of functions, including prey wrapping, prey capture, egg casings, and web framing [1]. Each task-specific silk is made up of a unique combination of proteins produced in one of seven different gland types [2, 3]. Despite making architecturally distinct prey-capture webs (Fig. 1), orb-web and cobweb weaving araneoid spiders possess homologous gland types, most of which produce silks with homologous functions. For example, major ampullate glands synthesize dragline fibers, tubuliform glands express proteins that form outer egg-casing fibers, and pyriform gland protein products are used to attach other fiber types to substrates by all araneoid spiders [1, 4]. Other gland types are homologous in morphology, but have diverged in some functions, such as minor ampullate glands which produce silk used as scaffolding fibers by most araneoids, but additionally functions to form auxiliary spirals in the orb-web versus contributing to prey-wrapping in cobweb weavers [5].

Major ampullate silk has been the primary focus of silk research because it is relatively easy to harvest and has tensile strength rivaling that of steel [6, 7]. However, all silk fibers have impressive and unique material properties [8]. For instance, minor ampullate silk of the orb-web weavers *Argiope trifasciata*, *Argiope argentata*, and *Nephila clavipes* have higher extensibility and lower strength than major ampullate silks from the same species, yet are equally tough [8–12]. Additionally, minor and major ampullate silks display different physical properties when wet. Supercontraction in water occurs with *A. trifasciata* and *Nephila inaurata* major ampullate silks, which allows them to become more extensible while maintaining their high strength [13]. However, when minor ampullate silks from the same species are exposed to water, supercontraction does not occur and the mechanical properties do not change [13]. Despite having consistent behaviors within species whether wet or dry, mechanical properties of minor ampullate silks varied more among species than did the properties of major ampullate silks [14, 15]. The consistency of minor ampullate silks’ mechanical properties when wet or dry, as well as their increased extensibility relative to draglines and greater variability among species, could allow for different applications of minor ampullate silk that would not be feasible for major ampullate silk. The material properties of minor ampullate silks made by cobweb weavers have yet to be measured, but given the different functions of this silk in cobweb weavers compared to in orb-web weavers, we predict divergence in the mechanical performance of these fibers.

The variation in physical properties between major and minor ampullate silks and among minor ampullate silks of different species likely results from sequence differences among their component proteins, termed spidroins (spider fibroins). Spidroins are extremely large (>200 kDa) and are made almost entirely of a highly repetitive region, which is flanked by short, conserved carboxy (C)- and amino (N)-terminal regions [16–21]. The repetitive region of many spidroins contain numerous subrepeats of short amino acid sequences, called motifs [2]. Motifs found frequently in spidroin repetitive regions include GGX (where X refers to a subset of amino acids), (GA)_n_ where *n* ≥ 2, A_n_ where *n* ≥ 4, and GPG [2, 18]. A_n_ and (GA)_n_ form β-sheets, GGX forms 3_1_-helices, and GPG forms β-spirals [2, 14, 18, 22–26]. Secondary structures of β-sheets likely confer strength, and β-spirals contribute to elasticity [18].

Major ampullate silks of all araneoid spiders examined thus far are made up of two proteins, MaSp1 and MaSp2, which are distinguished by a high GPG content in the latter but not the former [27, 28]. Similarly, *N. clavipes* minor ampullate silk is made up of two proteins, MiSp1 and MiSp2, although they are not as distinctly different from each other as MaSp1 and MaSp2 [29]. All described minor ampullate spidroins (generalized as MiSp) have consensus repeats that are primarily made up of A_n_, (GA)_n_ and GGX motifs disrupted by serine and threonine-rich “spacer” regions [2, 14, 18, 29, 30]. The functions of the spacer regions are not well understood, but artificially made recombinant proteins composed solely of spacer regions tend to form oligomers and only recombinant MiSp proteins that include at least one spacer, repetitive region, and the C-terminal domain can form fibers [31]. Both MaSp1 and MiSp are predicted to be composed of β-sheets connected by helical regions and amorphous chains [2, 14, 18]. The higher tensile strength of major ampullate silk may result from longer β-sheets made up of A_n_ repeats, in contrast to shorter β-sheets made up of A_n_ and (GA)_n_ repeats in minor ampullate silk [14].

The large size and repetitiveness of spidroins make spidroin-encoding genes extremely difficult to sequence completely. The only full-length sequence of *MiSp* known is for *Araneus ventricosus* [30]. Furthermore, even partial *MiSp* sequences have been characterized from only a few orb-web weavers [29, 30]. The full length sequence shows that MiSp repeat units are not as similar to each other as are the repeats of MaSp1 or MaSp2 repeats; and that *A. ventricosus* MiSp possesses exactly two spacers that interrupt the glycine and alanine rich motifs [30]. The generality of these features for other MiSp-encoding sequences needs to be tested.

The only known sequences of *MiSp* in a cobweb weaver are partial sequences from *Latrodectus hesperus* [5, 32]. The known repetitive regions are similar between *L. hesperus* and orb-web weavers’ MiSp, with the main difference being length of spacers [30, 32]. However, because cobweb weavers use minor ampullate silks for prey wrapping, while orb-web weavers use them in the auxiliary spiral of their web [5], we predict different selective pressures on cobweb weaving MiSp that could consequently alter secondary structure and physical properties.

Here, we characterize complete or nearly complete *MiSp* sequences from five cobweb weaving spider species: *L. hesperus*, *L. tredecimguttatus*, *L. geometricus*, *Steatoda grossa*, and *Parasteatoda tepidariorum* (Theridiidae). We also measured mechanical properties of minor ampullate silks in *L. hesperus, L. geometricus*, and *S. grossa*. Our goals were to determine the extent of variation in MiSp sequences and minor ampullate silk properties within cobweb weaving spiders, compare this variation to that found in orb-web weaving spiders, and identify any relationship between sequence and material property variation across both groups. We found evidence for at least two *MiSp* loci in each cobweb weaving species, and that one copy is more highly expressed than the other in minor ampullate glands in *L. hesperus* and *L. geometricus*. Phylogenetic analyses suggested extensive gene duplication and concerted evolution of MiSp-encoding loci contributed to variation in MiSp sequences. Finally, we found significant differences in mechanical properties among minor ampullate silks that were partially explained by variation in MiSp sequences.

## Results

### Multiple loci encode MiSp in cobweb weavers

We employed a series of molecular experiments to identify MiSp-encoding sequences in our five species of cobweb weavers. Additional file 1: Figure S1 provides an overview of these various methods.

#### *L. hesperus*

Shotgun sequencing of an *L. hesperus MiSp*-positive fosmid genomic clone resulted in the assembly of three contiguous sequences (contigs). One ~13 kilobase (kb) contig contained a complete open reading frame (ORF) predicted to encode MiSp (6549 base pairs, bp) and 4689 bp upstream and 2183 bp downstream flanking sequence. Amplification and cloning of *L. hesperus* genomic DNA with primers designed from the 3’UTR of a *MiSp* cDNA and an N-terminal encoding cDNA from this species resulted in an almost complete ORF (5568 bp) and 85 bp of 3’UTR, which was 98.8% identical to the published *MiSp* cDNA (HM752571) over a 983 bp alignment and 97.7% identical to the published *MiSp* N-terminal cDNA (HM752570) over a 903 bp alignment. In contrast to the similarity between the amplified gene and the cDNAs, the fosmid clone and the amplified gene were distinct. Pairwise differences between the fosmid clone and the amplified gene were 4.5% at the N-terminal encoding end (975 bp alignment) and 20.3% at the C-terminal/ 3’ UTR end (960 bp alignment). The repetitive regions were too divergent to align. We designated the fosmid clone as *Lh* MiSp variant 1 (*Lh* MiSp_v1) and the amplified gene as *Lh* MiSp variant 2 (*Lh* MiSp_v2; see Table 1 for accession numbers).

**Table 1:** Spidroins included in phylogenetic analyses of N- and C-terminal sequences

Spidroin name	Species	Family	N-term accession	C-term accession
*Bc* fibroin1	*Bothriocyrtum californicum*	Ctenizidae	HM752562	EU117162
*Kh* MaSp1	*Kukulcania hibernalis*	Filistatidae	HM752563	
*Dc* MaSp	*Diguetia canities*	Diguetidae	HM752564	HM752565
*Dc* MaSp-like	*Diguetia canities*	Diguetidae	HM752566	HM752567
*Ds* MaSp2	*Deinopis spinosa*	Deinopidae	HM752568	DQ399328, DQ399329^a^
*Aap* MaSp	*Agelenopsis aperta*	Agelenidae	HM752573	AAT08436
*Ud* MiSp	*Uloborus diversus*	Uloboridae	HM752574	ABD61597
*Mg* MiSp	*Metepeira grandiosa*	Araneidae	HM752575	HM752569
*Lh* MiSp	*Latrodectus hesperus*	Theridiidae	HM752570	HM752571
*Aap* TuSp1	*Agelenopsis aperta*	Agelenidae	HM752576	HM752572
*Aa* TuSp1	*Argiope argentata*	Araneidae	HM752577	AY953071
*Ab* TuSp1	*Argiope bruennichi*	Araneidae	AB242144	AB242144
*Nct* TuSp1	*Nephila clavata*	Nephilidae	AB218974	AB218973
*Lh* TuSp1	*Latrodectus hesperus*	Theridiidae	DQ379383	AY953070
*At* MaSp2	*Argiope trifasciata*	Araneidae	DQ059136S1	DQ059136S2
*Nc* MaSp2	*Nephila clavipes*	Nephilidae	EU599240	AY654297
*Ni* MaSp2	*Nephila inaurata madagascariensis*	Nephilidae	DQ059135	AF350278
*Nc* MaSp1a	*Nephila clavipes*	Nephilidae	EU599238	AY654292
*Nc* MaSp1b	*Nephila clavipes*	Nephilidae	EU599239	AY654291
*Lh* MaSp1	*Latrodectus hesperus*	Theridiidae	EF595246	EF595246
*Lh* MaSp2	*Latrodectus hesperus*	Theridiidae	EF595245	EF595245
*Lg* MaSp1	*Latrodectus geometricus*	Theridiidae	DQ059133S1^b^	DQ059134
*Ea* MaSp	*Euprosthenops australis*	Pisauridae	AM259067	AJ973155
*Nc* Flag	*Nephila clavipes*	Nephilidae	AF027972^b^	AF027973
*Ni* Flag	*Nephila inaurata madagascariensis*	Nephilidae	AF218623S1	AF218623S2
*Av* Flag	*Araneus ventricosus*	Araneidae	AY945306	AY587193
*Lg* MaSp2	*Latrodectus geometricus*	Theridiidae	EU177657	AF350275
*Ds* fibroin1a	*Deinopis spinosa*	Deinopidae	JX978170	DQ399326
*Lh* AcSp1	*Latrodectus hesperus*	Theridiidae	JX978171	
*Lg* AcSp1	*Latrodectus geometricus*	Theridiidae	JX978180	JX978181
*Lh* MiSp_v1	*Latrodectus hesperus*	Theridiidae	KX584003	
*Lh* MiSp_v2	*Latrodectus hesperus*	Theridiidae	KX584020	
*Lg* MiSp_v1	*Latrodectus geometricus*	Theridiidae	KX584023	
*Lg* MiSp_v2	*Latrodectus geometricus*	Theridiidae	KX584024	
*Lt* MiSp_v1	*Latrodectus tredecimguttatus*	Theridiidae	KX584027	
*Lt* MiSp_v2	*Latrodectus tredecimguttatus*	Theridiidae	KX584026	
*Sg* MiSp_pseudo	*Steatoda grossa*	Theridiidae	KX584019	
*Sg* MiSp	*Steatoda grossa*	Theridiidae	KX584035	KX584021
*Pt* MiSp	*Parasteatoda tepidariorum*	Theridiidae	KX584055	KX584022
*Ncr* PySp	*Nephilengys cruentata*	Nephilidae		GU062417
*Nc* PySp	*Nephila clavipes*	Nephilidae		GQ980330
*Nc* PySp2	*Nephila clavipes*	Nephilidae		HM020705
*Lh* PySp	*Latrodectus hesperus*	Theridiidae		FJ973621
*Ud* AcSp1	*Uloborus diversus*	Uloboridae		DQ399333
*Na* MiSp	*Nephila antipodiana*	Nephilidae		DQ399324
*Nc* MiSp1	*Nephila clavipes*	Nephilidae		AF027735
*Ncr* MiSp	*Nephilengys cruentata*	Nephilidae		EF638447
*Ds* MiSp	*Deinopis spinosa*	Deinopidae		DQ399324
*Ad* MiSp	*Araneus diadematus*	Araneidae		U47853
*Av* MiSp	*Araneus ventricosus*	Araneidae		JX513956.1
*Pb* MiSp	*Parawixia bistriata*	Araneidae		GQ275358
*Ad* MaSp1	*Araneus diadematus*	Araneidae		U47854
*Ad* MaSp2a	*Araneus diadematus*	Araneidae		U47855
*Ad* MaSp2b	*Araneus diadematus*	Araneidae		U47856
*Ncr* MaSp_like	*Nephilengys cruentata*	Nephilidae		EF638446
*Na* MaSp1	*Nephila antipodiana*	Nephilidae		DQ338461
*Ud* MaSp1	*Uloborus diversus*	Uloboridae		DQ399331
*Ud* MaSp2a	*Uloborus diversus*	Uloboridae		DQ399334
*Ud* MaSp2b	*Uloborus diversus*	Uloboridae		DQ399335
*Ncr* Flag	*Nephilengys cruentata*	Nephilidae		EF638444
*Ncr* TuSp	*Nephilengys cruentata*	Nephilidae		EF638445
*Ds* TuSp1	*Deinopis spinosa*	Deinopidae		AY953073
*Ud* TuSp1	*Uloborus diversus*	Uloboridae		AY953072
*Na* TuSp1	*Nephila antipodiana*	Nephilidae		DQ089048

^a^DQ399329 contains the *Ds* MaSp2a C-terminal sequence and DQ399328 the *Ds* MaSp2b C-terminal sequence

^b^DQ059133S1 and AF027972 were edited to include corrections outlined by [73]

Primer sets designed to be specific to *Lh* MiSp_v1 versus *Lh* MiSp_v2 successfully amplified genomic DNA of three single individuals. Variant-specific PCR products were 99.3% identical to the targeted variant, but only 86.3% identical to each other on average at the 616 bp C-terminal/ 3’UTR region. Sequence chromatograms of *Lh* MiSp_v1 and MiSp_v2 amplifications exhibited double peaks in both directions in at least one individual (10 double peaks in one of three individuals amplified with *Lh* MiSp_v1 specific primers and one double peak in all three individuals amplified with *Lh* MiSp_v2 specific primers). The pattern of double peaks in one individual but not others for the *Lh* MiSp_v1 specific PCR product is consistent with allelic variation for *Lh* MiSp_v1 and confirms that *Lh* MiSp_v1 and *Lh* MiSp_v2 represent separate genomic loci.

#### *L. tredecimguttatus* and *L. geometricus*

**Fig. 2:**
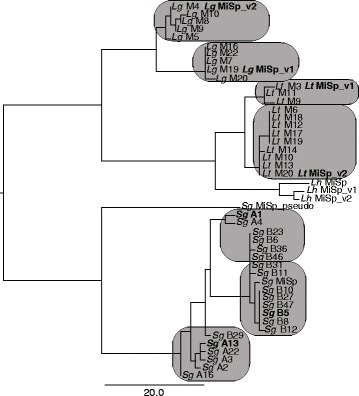
Neighbor joining tree for N-terminal MiSp encoding sequences demonstrates variation within and among species. Variants are shown for *L. hesperus* (*Lh*) and TOPO clones from *L. tredecimguttatus* (*Lt*), *L. geometricus* (*Lg*), and *S. grossa* (*Sg*). Units are number of substitutions. *Latrodectus* clones are arbitrarily numbered, except that *Lt* M1-7 and *Lg* M1-5 resulted from amplification with primers designed from *L. hesperus*, while higher clone numbers resulted from amplification with species-specific primers. *S. grossa* TOPO clones resulted from two separate PCR reactions, which are indicated here as “A#” and “B#”. Completely sequenced clones are indicated by *bolded* names. See Table 1 and Additional file 1: Table S4 for accession numbers. *Boxes* indicate distinct clusters identified in neighbor joining trees based on N-terminal sequences including adjacent repetitive sequences and in C-terminal and adjacent repetitive region encoding sequences (Additional file 1: Figures S2 and S3)

Cloning and end sequencing of nearly complete *MiSp* PCR products from genomic DNA of single individuals of *L. tredecimguttatus (Lt)* and *L. geometricus* (*Lg*) resulted in 11 unique *Lg* sequences (11 clones) and 11 unique *Lt* sequences (12 clones). Within each of these species, end sequences clustered into two distinct groups (Fig. 2; Additional file 1: Figure S2). Average pairwise differences within a cluster were low for N-terminal and adjacent repetitive encoding sequences: 0.9% (0–1.2%) and 2.0% (1.2–3.5%) for *Lt*; 0.7% (0–1.6%) and 1.0% (0.2–1.5%) for *Lg*. Average pairwise differences between clusters for the same region were much higher: 6.4% (4.5–7.6%) for *Lt* and 11.6% (5.3–14%) for *Lg*. A similar pattern was found for C-terminal and adjacent repetitive encoding sequences, except divergence among sequences was higher; average pairwise distances within clusters were 0.7% (0–1.5%) and 8% (4.5–15%) for *Lt*, and 2.8 (0–9.1%) and 6.0 (0.2–12.6%) for *Lg*; versus average pairwise distances between clusters were 10.2% (2.3–19.5%) for *Lt* and 52% (42.1–56.6%) for *Lg*.

Because these species are diploid, we expect no more than two alleles per locus within a single individual. Thus each species may possess as many as six loci encoding MiSp (11 variants identified in each species). However, because some PCR or cloning error could produce slight variants among sequences (despite the use of a proofreading enzyme) we conservatively considered the two distinct clusters to represent separate loci and completely sequenced one representative from each cluster (Fig. 2). We designated the completely sequenced clones from *L. tredecimguttatus* as *Lt* MiSp_v1 and *Lt* MiSp_v2, and the completely sequenced clones from *L. geometricus* as *Lg* MiSp_v1 and *Lg* MiSp_v2. Designation of MiSp variants with 1 or 2 is not intended to imply orthology among species, but simply reflects different genomic loci within species.

#### *S. grossa*

TOPO cloning and end sequencing of an ~5000 bp *S. grossa* PCR product that was generated with primers targeting complete *MiSp* resulted in ten identical clones from a single individual. When translated, these ~800 bp end sequences aligned well with our *Latrodectus* MiSp sequences, but contained numerous stop codons. This variant is likely a non-functional pseudogene, designated *Sg* MiSp_pseudo.

PCR amplification using primers designed from our ~3 kb C-terminal encoding *S. grossa* MiSp cDNA (designated *Sg* MiSp) failed to find any further variants. In contrast, TOPO cloning and sequencing of 20 N-terminal and adjacent repetitive MiSp encoding PCR products for a single *S. grossa* individual resulted in 20 unique sequences, each different from our ~500 bp N-terminal encoding cDNA. The clones (designated by clone numbers) grouped into three clusters (Additional file 1: Figure S3); within a cluster pairwise differences among sequences were <0.9% on average (0.13–1.5%) while between clusters pairwise differences were much greater (average pairwise distance = 10%). Nucleotide differences within a cluster could reflect alleles or multiple loci, but could also be attributable to PCR error because amplification was performed with *Taq* polymerase alone, which does not have proofreading capability, in contrast to the high fidelity polymerases used to amplify the *Latrodectus* species (see Methods). Conservatively, the three clusters represent three true variants. Thus there is a minimum of two loci that encode MiSp in *S. grossa*.

#### *P. tepidariorum*

We found partial length N and C-terminal MiSp-encoding cDNAs from *P. tepidariorum* (designated *Pt* MiSp, see Table 1 for accessions) that were nearly identical (>98%) to the 5’ and 3’ ends, respectively, of an Augustus-predicted gene model on Scaffold 853 in the publicly available genome. This gene model was predicted to span 10292 bp, but included four gaps (4353 bp of assembled sequence, 5939 bp of unknown). Characterization of a TOPO clone containing an ~2.3 kb PCR product for *P. tepidariorum MiSp* (KX584004) resulted in a sequence that was 100% identical to the 104 bp of overlap with the C-terminal encoding region of Scaffold 853 and filled 2236 bp of the last gap. We refer to the combination of our TOPO clone with Scaffold 853 as *Pt* MiSp variant 1 (*Pt* MiSp_v1). The fully sequenced TOPO clone included a 1175 bp long intron with canonical donor and acceptor sites identified by aligning with our C-terminal encoding cDNA using SPIDEY [33]. We also found BLASTN alignments of this 2.3 kb TOPO clone to three other scaffolds (954, 3280, and 3758) that may represent additional MiSp-encoding loci. The C-terminal encoding region of our TOPO clone aligned with 91% identity on Scaffold 954. The intron sequence aligned with 95% identity to Scaffold 3280 and 99% identity to Scaffold 3758. The repeat region flanking the intron (including 100 bases of intron) aligned with 89% identity to Scaffold 3280 and 90% identity to Scaffold 3758. The presence of two C-terminal encoding sequences that differ by 9% and are placed on separate scaffolds suggests that at least two loci encode MiSp in *P. tepidariorum*.

### MiSp loci are expressed at different rates in cobweb weaving spiders

In *L. geometricus* and *L. hesperus*, both completely sequenced loci (*MiSp_v1* and *MiSp_v2)* are highly transcribed in minor ampullate glands (2.4–44.7% of all reads in minor ampullate gland RNA-seq libraries aligned to *MiSp_v1* or *MiSp_v2*) and transcribed at low levels in other tissue types (e.g. <1% of all reads in major ampullate gland RNA-seq libraries aligned to *MiSp_v1* or *MiSp_v2*, Table 2). *MiSp_v2* transcripts are more abundant than *MiSp_v1* in both *L. geometricus* and in *L. hesperus* minor ampullate glands (Table 2). The ratio was consistent for all tissue types in *L. hesperus,* however, ratios were much more variable in *L. geometricus* between libraries of minor ampullate glands and even more so among tissues (Table 2).

**Table 2:** *MiSp* transcript abundance based on read counts in minor ampullate, major ampullate, and total silk gland RNA-seq libraries

	*L. geometricus*					*L. hesperus*				
RNA-seq Library	Total reads^a^	*MiSp_v2*	*MiSp_v1*	Ratio^c^	Ratio 3’^d^	Total reads^b^	*MiSp_v2*	*MiSp_v1*	Ratio^c^	Ratio 3’^d^
Minor 1	4832818	275164	57912	4.8	2.6	11679960	5289870	1487650	3.6	3.6
Minor 2	20640831	3496061	564983	6.2	4.2	11148437	4919531	1443331	3.4	3.4
Total Silk	8101748	61016	72774	0.8	0.9	15093424	572185	183028	3.1	4.3
Major 1	13978630	1943	4493	0.4	0.3	6116968	68	24	2.8	2.7
Major 2	13636997	69	9	7.7	5.5	9574596	315	85	3.7	4.4

See Table 1 for accession numbers

^a^ Raw RNA-seq reads are available on NCBI’s SRA database: SRR1539569

^b^ Raw RNA-seq reads are available on NCBI’s SRA database: SRR1539570

^c^ Ratio of *MiSp_v2* to *MiSp_v1* based on counts of reads aligned to the entire sequences

^d^ Ratio of *MiSp_v2* to *MiSp_v1* based on counts of reads aligned to the 3’-most 500 bases of coding sequences. See Methods for rationale

### Variable genomic structure of MiSp

All *MiSp* genes characterized from *Latrodectus* lacked introns interrupting coding sequence. While only *L. hesperus MiSp_v1* included both the start and stop codons, the other *MiSp* sequences missed no more than 300 bp of the N or C-terminal coding regions on either end. Our *P. tepidariorum MiSp* genomic TOPO clone that includes the C-terminal encoding region has an intron of 1175 bp. For comparison, orb-weaver *A. ventricosus MiSp* has a large, single intron of 5628 bp [30].

Inspection of the *L. hesperus* fosmid genomic clone revealed a TATA box approximately 50 bases upstream of the full-length *L. hesperus* MiSp coding region. The nucleotide motif CACG found in all previously characterized spidroin genes was also found in *L. hesperus* MiSp, 11 bases upstream of the TATA box. The *P. tepidariorum* sequence on Scaffold 853 in the i5k genome possessed a TATA box 63 bases upstream of the MiSp coding region and a CACG motif 10 bases upstream of the TATA box. The *A. ventricosus* sequence has a TATA box 63 bases upstream of the full-length MiSp coding region, and a CACG motif 30 bases upstream of the TATA box [30]. Polyadenylation signals were found 110 bases downstream of the stop codon for *L. hesperus MiSp_v1*, 242 bases downstream of the stop for *P. tepidariorum MiSp_v1,* and 82 bases downstream of the stop for *A. ventricosus MiSp* [30].

### MiSp amino acid variation within and among species

Cobweb weaver MiSp length is highly variable within and among species (815 aa to 2751 aa, Additional file 1: Figure S4). For comparison, the completely sequenced *L. hesperus* MaSp1 is 3129 aa and MaSp2 is 3779 aa, and *A. ventricosus* MiSp is 1,766 aa [30]. Glycine, alanine, and serine are the three most abundant amino acids for all MiSp variants, showing similar composition percentages to *L. hesperus* MaSp1 and MaSp2. Serine is found in both the spacer regions and the repeat regions, while glycine and alanine are predominantly found in the repeat region only. Proline is found in very low abundance for both *L. hesperus* MiSp variants*,* both *L. tredecimguttatus* MiSp variants, *L. geometricus* MiSp_v2, and *P. tepidariorum* MiSp_v1, but is more abundant in *L. geometricus* MiSp_v1 and is the fourth most abundant amino acid in *S. grossa* MiSp, surpassing *L. hesperus* MaSp2 proline composition (Additional file 1: Figure S4). Codon usage is variable among MiSp loci and species, but there is an overall bias toward A or T in the third position of the codon, especially for alanine and glycine (Additional file 1: Table S1).

**Fig. 3:**
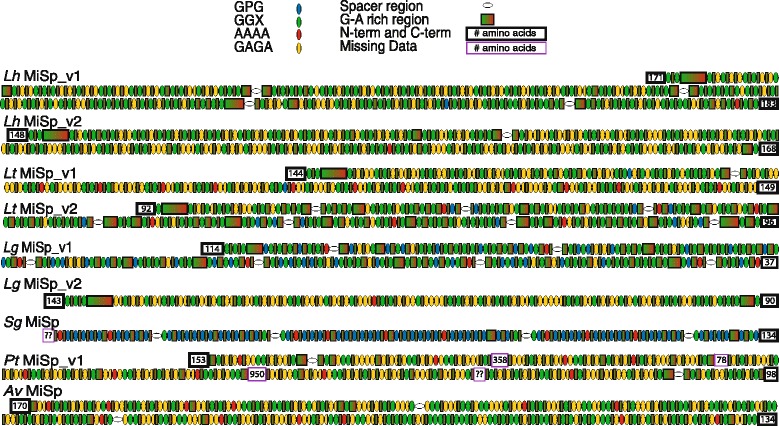
Arrangement of amino acid motifs in MiSp sequences of cobweb and orb-web weaving spiders. *Latrodectus* and *S. grossa* accession numbers are in Table 1. *Lh*, *L. hesperus*; *Lt*, *L. tredecimguttatus*; *Lg*, *L. geometricus*; *Sg*, *S. grossa*; *Pt*, *P. tepidariorum* based on combining the incompletely assembled MiSp-encoding region from Scaffold 853 of the i5K genome with an ~2.3 kb TOPO-cloned PCR product, KX584004; *Av*, *A. ventricosus,* JX513956.1 [30]. Lengths of missing amino acid sequences in *Pt* MiSp_v1 are based on the length of gaps in Scaffold 853 of the i5K genome, assuming no introns are present within those gaps. The last gap was predicted to be 1782 bp long (594 aa if no intron) in Scaffold 853, but our TOPO clone added 2236 bp, including a 1175 bp intron, which was longer than the predicted gap. The repetitive encoding region at the 3’ end of our TOPO clone did not overlap with the encoding region present in the i5K genome prior to the last gap

Despite gross similarities in MiSp amino acid composition among species, arrangement of amino acids into motifs is more variable. *L. hesperus* MiSp variants, *L. tredecimguttatus* MiSp variants, *L. geometricus* MiSp_v2, and *P. tepidariorum* MiSp_v1 contain mostly (GA)_n_ and GGX motifs, with GPG being nonexistent. However, GPG is prevalent in both *L. geometricus* MiSp_v1 and *S. grossa* MiSp (Fig. 3). In contrast to MaSp1 and MaSp2, which have ensemble repeat units ~30–50 aa long that are nearly identical, there are multiple shorter, more variable ensemble repeats that describe MiSp sequences (Additional file 1: Table S2). Tandem repeats range in length from 7 to 569 aa, repeated in the protein anywhere from 2 to 28 times, and with amino acid sequence identity ranging from 81% to 97% (Additional file 1: Table S2). Modularity of ensemble repeats is highly variable among and within species, with *L. hesperus* MiSp_v1*, L. tredecimguttatus* MiSp_v2, *L. geometricus* MiSp_v1, and *S. grossa* MiSp having a higher order repeat unit (>160 aa repeated at least once) and *L. hesperus* MiSp_v2, *L. tredecimguttatus* MiSp_v1, *L. geometricus* MiSp_v2, and *P. tepidariorum* MiSp_v1 having multiple short repetitive units only (24 to 30 aa repeated >10 times).

**Fig. 4:**
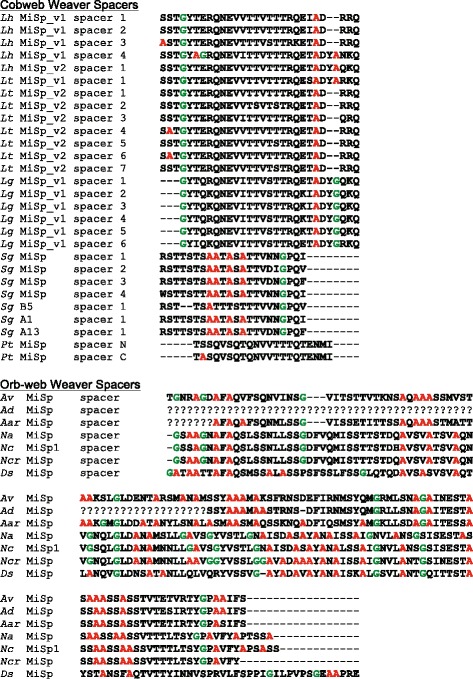
Comparison of theridiid (*top*) and orb-web weaver (*bottom*) MiSp spacers. Theridiid MiSp spacers are aligned for *L. hesperus* (*Lh*), *L. tredecimguttatus* (*Lt*), *L. geometricus* (*Lg*), *S. grossa* (*Sg*), and *P. tepidariorum* (*Pt*). Orb-web weaver MiSp spacers aligned for *A. ventricosus* (*Av*), *A. diadematus* (*Ad*), *Argiope argentata* (*Aar*), *Nephila antipodiana* (*Na*), *N. clavipes* (*Nc*), *Nephilengys cruentata* (*Ncr*), and *Deinopis spinosa* (*Ds*) following [30]. See Table 1 and Additional file 1: Table S4 for accession numbers. Note that the sequence of spacers in the *Pt* MiSp N and C-terminal encoding cDNAs shown here are identical to spacers found in *Pt* MiSp_v1

The number and arrangement of spacers is also highly variable among cobweb weaver MiSp. Some MiSp variants lack spacers while others have up to seven spacers. Spacers are varied in their distribution within a monomer, ranging from 100 aa to 400 aa away from each other in MiSp proteins with more than one spacer (Fig. 3). Spacers are easily distinguished from the repetitive motifs in their amino acid composition (rich in threonine, serine, and valine), near perfect repetition within a MiSp protein, and similar length across species (Fig. 4). Furthermore, with the exception of *S. grossa* MiSp, spacers are much more hydrophilic than the repetitive region (Additional file 1: Figure S5).

### Material properties of minor ampullate spidroins correlate with MiSp sequence variation

**Fig. 5:**
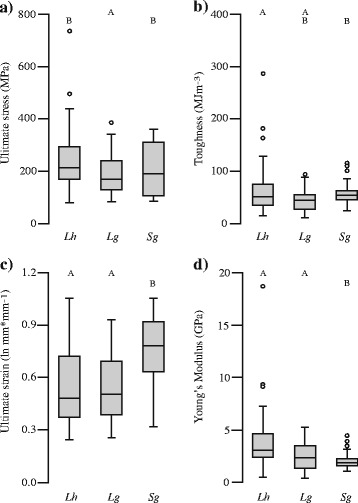
Minor ampullate silk fiber tensile properties differ among three cobweb weaving species. Boxplots show differences among *L. hesperus* (*Lh*)*, L. geometricus* (*Lg*), *and S. grossa* (*Sg*) for **a)** strength (MANOVA, F_2,147_ = 8.9, *p* < 0.001); **b)** toughness (MANOVA, F_2,147_ = 6.8, *p* = 0.002); **c)** extensibility (MANOVA, F_2,147_ = 13.3, *p* < 0.001); and **d)** Young’s Modulus (MANOVA, F_2,147_ = 12.9, *p* < 0.001). Open circles are outliers that exceed the range of the interquartile distance by 1.5 times. *Different letters* (e.g. A vs B) above *boxplots* indicate significant pairwise differences according to Tukey’s Test using a harmonic mean sample size of 50 and α = 0.05

We found significant differences in each of four tensile properties of minor ampullate silk fibers measured for three cobweb weaving species (Fig. 5). These properties were tensile strength, the amount of force applied to break a single silk fiber, accounting for the diameter of the fiber; extensibility, the proportion of the original fiber length that the fiber was extended at breakage; stiffness or Young’s modulus, the initial slope of the force-extension curve (initial resistance to force); and toughness, the energy required to break the fiber (see Methods for details). Post-hoc Tukey’s test revealed that minor ampullate silk from *L. geometricus* had the lowest tensile strength (Fig. 5). Minor ampullate silk from *S. grossa* was significantly more extensible and less stiff than the other two species (Fig. 5).

Minor ampullate silks from the cobweb weavers are generally weaker, less stiff, and less tough than orb-web weavers, but more extensible (Table 3). We found a positive, but non-significant, relationship between the proportion of the GPG motif in MiSp and the extensibility of minor ampullate silk fibers among the five species with both published MiSp sequences and material properties (adjusted *R* ^*2*^ = 0.53, *p* = 0.10; Additional file 1: Figure S6). However, when accounting for phylogenetic relationships and evolutionary distances among species using phylogenetic independent contrasts, we found a significant positive relationship between GPG proportion and extensibility (adjusted *R* ^*2*^ = 0.92, *p* = 0.006), due to the extensive change in GPG and extensibility on the relatively short *S. grossa* branch (see Additional file 1: Figure S6 for the effect of varying branch lengths). This pattern of higher GPG content contributing to extensibility parallels patterns found for major ampullate silks, although for major ampullate silks higher GPG content results from higher expression of MaSp2, rather than sequence differences among orthologs [2, 14, 18, 22–25].

**Table 3:** Properties of minor ampullate silk for cobweb and orb-web weaving species

Species	Diameter (μm)	Young’s Modulus (GPa)	Strength (MPa)	Extensibility (ln mm/mm)	Toughness (J/cm^3^)	GPG (%)
*Latrodectus hesperus*	1.12 ± 0.47	3.9 ± 2.9	245.4 ± 120.5	0.57 ± 0.02	66.7 ± 46.4	0.07 ^g^
*Latrodectus geometricus*	1.09 ± 0.29	2.6 ± 1.3	174.4 ± 17.6	0.54 ± 0.02	43.6 ± 20.1	3.48 ^h^
*Steatoda grossa*	1.07 ± 0.26	2.1 ± 0.7	251.3 ± 106.2	0.74 ± 0.02	57.3 ± 20.7	12.79 ^i^
*Nephila clavipes* ^a^	2.5 ± 0.2	3.0 ± 0.6	346 ± 53	0.30 ± 0.15		0 ^j^
*Nephila clavipes* ^b^	2.5		960 ± 50	0.25 ± 0.03		0 ^j^
*Nephila inaurata* ^c^	1.8 ± 0.1	11.2 ± 0.7	1500 ± 200	0.46 ± 0.05	300 ± 50	
*Argiope trifasciata* ^d,e^	0.69 ± 0.10	8.9 ± 0.05	751.5 ± 40	0.44 ± 0.04	150 ± 12	
*Argiope trifasciata* ^c^	1.8 ± 0.1	10 ± 0.4	1040 ± 6	0.45 ± 0.02	240 ± 20	
*Argiope argentata* ^e^	1.1 ± 0.7	10.6 ± 1.2	923 ± 154	0.33 ± 0.03	137 ± 22	
*Araneus diadematus* ^f^				0.29		0 ^k^
*Araneus gemmoides* ^b^	2.0		1400 ± 100	0.22 ± 0.07		

Material properties collected from ^a^[10], ^b^[15], ^c^[13], ^d^[9], ^e^[8], ^f^[74]

Proportion of MiSp that is made up of the motif GPG was averaged across sequences described for each species: ^g^ *Lh* MiSp_v1, MiSp_v2; ^h^ *Lg* MiSp_v1, MiSp_v2; ^i^ *Sg* MiSp: C-terminal encoding cDNA (KX584021); ^j^ AF027735; ^k^ U47853

### Phylogenetic relationships of spidroins among orb-web and cobweb weaving species suggest multiple duplication events

**Fig. 6:**
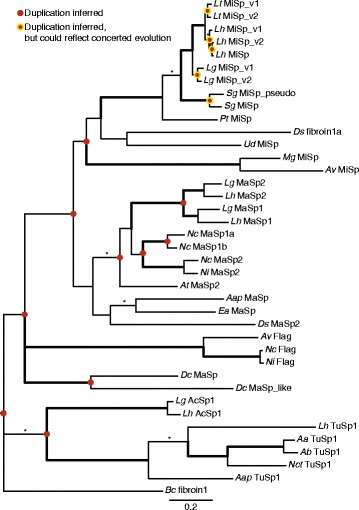
Bayesian tree for combined N- and C-terminal spidroin-encoding nucleotide sequences. See Table 1 for accessions. *Bold branches* are supported by > 50% bootstrap parsimony and > 95% posterior probability in all analyses of combined N- and C-terminal sequences (see Additional file 1: Figure S7). Asterisks (*) indicate > 95% posterior probability in Bayesian analyses of N- and C-terminal sequences. Units are substitutions per site. The tree is rooted with *Bc* fibroin1. Duplications inferred by reconciliation with the species trees in Fig. 1 are shown as *red dots* at nodes. The *red dots* with *yellow* outlines indicate that concerted evolution of terminal encoding regions could also explain the observed relationships

Spidroin N- and C-terminal domains and encoding nucleotides demonstrate MiSp sequences found in eight species form a monophyletic group that is usually recovered as sister to a clade of MaSp1 and MaSp2 sequences (Fig. 6, Additional file 1: Figure S7). Theridiid MiSp consistently group together with strong support (Fig. 6, Additional file 1: Figure S7). Within theridiids, MiSp sequences from *Latrodectus* species group together, *S. grossa* MiSp variants form a sister clade to *Latrodectus* MiSp variants, and *P. tepidariorum* MiSp is sister to *Latrodectus* and *Steatoda* MiSp variants. Within *Latrodectus, L. geometricus* MiSp variants form a sister clade to *L. hesperus and L. tredecimguttatus* MiSp variants; these patterns are congruent with species relationships [34, 35] (Fig. 6). However, MiSp variants group together within a species, suggesting independent duplication events within each species as inferred by reconciling our gene trees with the species trees in Fig. 1 (Fig. 6). Alternatively, this pattern could be explained by concerted evolution of terminal-encoding regions within each species, which we detail in the Discussion.

**Fig. 7:**
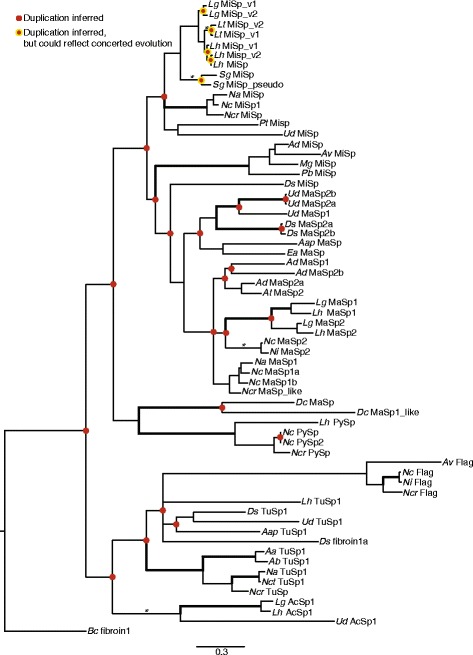
Bayesian tree for C-terminal spidroin-encoding nucleotide sequences. See Table 1 for accessions. *Bold branches* are supported by > 50% bootstrap parsimony and > 95% posterior probability in all analyses of C-terminal sequences (see Additional file 1: Figure S8). Asterisks (*) indicate > 95% posterior probability in Bayesian analyses of C-terminal sequences. Units are substitutions per site. The tree is rooted with *Bc* fibroin1. Duplications inferred by reconciliation with the species trees in Fig. 1 are shown as *red dots* at nodes. The *red dots* with *yellow* outlines indicate that concerted evolution of terminal encoding regions could also explain the observed relationships

Outside of Theridiidae, relationships among MiSp sequences are more ambiguous and do not necessarily reflect species relationships. For instance, molecular and morphological characters strongly support monophyly of the Araneoidea (represented here by Theridiidae, Nephilidae, and Araneidae) to the exclusion of Deinopidae and Uloboridae [36–39]. In contrast, N- and C-terminal domains sometimes place deinopid and uloborid MiSp sister to theridiid MiSp, and sometimes place araneid MiSp sequences sister to theridiid MiSp sequences (Fig. 6, Additional file 1: Figure S7). The former placement is consistent with an ancient duplication of a MiSp-encoding locus in the common ancestor of Araneoidea, Deinopidae, and Uloboridae (Fig. 6). C-terminal domains and encoding nucleotides for MiSp from 14 species generally show the same patterns within Theridiidae, but theridiid MiSp sequences tend to group with nephilid and deinopoid MiSp sequences to the exclusion of araneid MiSp sequences, consistent with multiple ancient duplication events (Fig. 7, Additional file 1: Figure S8). Relationships among other spidroins are consistent with previous findings that gland-specific spidroins tend to group together, with the exception of major ampullate spidroins [17, 32, 40]. Overall, we inferred 24–30 duplication events within the spidroin gene family, depending on which C-terminal gene tree was used for reconciliation (Additional file 1: Figure S8).

## Discussion

Our characterization of complete or almost complete encoding sequences for the minor ampullate spidroin (MiSp) in multiple cobweb weaving spider species (Theridiidae) demonstrate the presence of at least two *MiSp* loci in each species. The substantial variation of MiSp sequences within and among species and its relationship to variation in material properties has multiple implications for molecular evolution, spider ecology, and biomimetic applications through recombinant DNA technology.

Two MiSp-encoding loci have also been documented in the golden orb-weaver spider, *Nephila clavipes* (Nephilidae), and therefore the presence of multiple MiSp-encoding loci in spider genomes most likely dates minimally back to the common ancestor of theridiids and nephilids (e.g. Araneoidea). Our reconciliation of gene trees with species trees suggests even older duplication events (Fig. 7). However, the maintenance of two loci appears to involve complex molecular evolutionary processes including intergenic concerted evolution of the loci within species, and multiple gains and losses of individual *MiSp* copies. Although our reconciliation analyses inferred independent duplication events within each of our cobweb weaving species, the grouping of *MiSp* loci within species based on the terminal domains (Figs. 6 and 7), could also result from intergenic concerted evolution of the N- and C-terminal encoding regions. We favor the latter hypothesis because of the dramatic differences in the repetitive region within some species (Fig. 3). Concerted evolution of the N and C-terminal encoding regions could occur through non-homologous recombination between the loci, facilitated by their similar sequences, as proposed for the major ampullate spidroin paralogs, *MaSp1* and *MaSp2*, of multiple species [2, 32, 41–43]. Within theridiids, the rate of concerted evolution appears to be faster than speciation, since relationships among each species’ pair of *MiSp* loci reflect species relationships (Figs. 6 and 7). Intergenic concerted evolution of terminal-encoding regions could be favored by selection because these regions are involved in assembly of multiple MiSp monomers into polymers and the conversion of the protein complex from a liquid to a solid [31, 44]. Highly similar terminal regions may be necessary for polymers to form.

Outside of theridiids, relationships among MiSp N and C-terminal domains are not congruent with species relationships. The low posterior probabilities and bootstrap support for these incongruous relationships suggest there is limited phylogenetic signal retained in the MiSp C-termini at the distant time scale of divergence of theridiids from other araneoid families (~170 million years ago, [36, 38, 39]). However, the consistent grouping of *Nephila* and theridiid MiSp C-terminal domains to the exclusion of araneid MiSp C-terminal domains suggests that nephilid and theridiid MiSp sequences are derived from a different ancient copy than the araneid MiSp sequences, as supported by our reconciliation analyses (Figs. 6 and 7). The loss of functional *MiSp* loci through multiple single nucleotide mutations is further supported by the presence of a *MiSp* pseudogene in the *S. grossa* genome. Many additional losses were inferred by our reconciliation analyses (Additional file 1: Figure S8), but because of incomplete spidroin sampling for most species we do not feel confident in estimating the extent of spidroin gene loss.

We found extensive variation in the MiSp repetitive regions between loci within a genome and among species (Fig. 3). MiSp variants in *L. hesperus* and *L. tredecimguttatus, L. geometricus* MiSp_v2*, P. tepidariorum* MiSp_v1, *Nephila* MiSp 1 & 2, and araneid MiSp sequences are similar in terms of amino acid motif composition. *S. grossa* MiSp and *L. geometricus* MiSp_v1 are especially divergent, with the difference between variants within *L. geometricus* being extremely striking (Fig. 3). The divergent repeats have an increased proline content, which likely occurred independently in *S. grossa* MiSp and *L. geometricus* MiSp_v1, based on gene tree relationships (Figs. 6 & 7). The mutation of alanine or glutamine codons to a proline codon requires only a single base change and the GPG motif is frequently found as GPGA or GPGQ, indicating that it could have evolved from (GA)_n_ or (GQ)_n_ motifs, which are the most common motifs in all other MiSp sequences (Fig. 3). Intragenic concerted evolution could rapidly proliferate these mutations throughout a single gene as has been proposed for other spidroin paralogs [16, 17, 45, 46]. The near identity of spacer sequences within each of our cobweb weaver MiSp variants (Fig. 4) supports the hypothesis that intragenic concerted evolution is common, as also found in orb-web weaver MiSp [30, 31]. However, intragenic concerted evolution must be offset by some other molecular processes, since the remainder of the repetitive regions are not as homogenized as the spacers (Fig. 3). The simple amino acid motifs found in MiSp such as (GA)_n_ are encoded by microsatellite-like sequences and could thus be prone to high rates of slipped strand mispairing. It is also possible that excessive homogenization of MiSp repetitive regions adversely affects its function and is eliminated by selection.

The high proportion of the GPG amino acid motif in *S. grossa* MiSp correlates with higher extensibility in *S. grossa* minor ampullate silk fibers (Fig. 5c, Table 3). GPG content is probably not the only predictor of extensibility, however, since *Latrodectus* minor ampullate fibers were more extensible than orb-web weavers’ even though GPG content for *L. hesperus* is similar to the orb-web weavers. Furthermore, we did not find a significant difference in extensibility between *L. hesperus* and *L. geometricus* minor ampullate silk fibers despite *L. geometricus* MiSp_v1 having a relatively high percentage of GPG motifs (Fig. 3). The *MiSp_v2*: *MiSp_v1* transcript ratio in *L. geometricus* suggests that the GPG-rich MiSp_v1 is in lower abundance in *L. geometricus* (Table 2), which could explain why there is not a significant increase in extensibility of its minor ampullate silk fibers in comparison to *L. hesperus*. Cobweb weaving spiders use MiSp in their prey wrapping silks [5], and evolution of extensibility-conferring motifs could potentially allow for capturing different prey types by *S. grossa* compared to other cobweb weavers, although little is known about the ecology or diet of this species. It is also possible that *L. geometricus* modulates expression of its two MiSp-encoding loci in response to prey availability. Experimentally manipulating prey type has been shown to affect proline content of major ampullate fibers in *Nephila*, potentially as a result of plastic changes in relative expression levels of the proline-poor MaSp1 and the proline-rich MaSp2 [47, 48]. Variation among individuals in *MaSp1* and *MaSp2* transcript abundance has also been demonstrated for black widows [49]. Analogous experiments have not been done for minor ampullate fibers. However, the variation in *MiSp_v2*: *MiSp_v1* ratios that we found between *L. geometricus* individuals (Table 2) suggests plasticity of expression is possible.

Our six complete or nearly complete single exon *MiSp*-containing clones can also serve as templates for recombinant proteins. Due to the dramatic differences in length and amino acid content of some of the proteins encoded by these loci, it may be possible to spin artificial fibers with custom-made properties. For instance, the GPG-containing *L. geometricus* MiSp_v1 could be used to make more extensible fibers, while the longer alanine-rich *L. hesperus* MiSp_v1 may make stiffer fibers.

## Conclusions

We found that intragenic concerted evolution within MiSp-encoding genes likely led to rapid proliferation of proline replacements for alanine or glutamine in MiSp protein sequences independently in at least two species. For one species, the proliferation of proline coincides with higher extensibility of minor ampullate silks. This could allow cobweb weavers to access new prey types or the ability to modulate mechanical properties of their silks through altering expression levels of *MiSp* gene copies. Our multiple nearly complete *MiSp* sequences also provide various templates for tailored biomimetic applications through recombinant DNA technologies.

## Methods

### Taxon sampling

Individual *L. hesperus* were collected in Riverside, California and Tucson, Arizona. *L. geometricus* individuals were collected in San Diego, California. *L. tredecimguttatus* and *S. grossa* were obtained from SpiderPharm (Yarnell, Arizona). *P. tepidariorum* were obtained from a laboratory culture founded with spiders collected near Cologne, Germany and purchased from SpiderPharm.

### Sequencing

#### Genomic library clone

We screened an *L. hesperus* genomic library with PCR for clones containing *MiSp* (see [16] for library construction and screening protocols). Primers used in screening were designed from the C-terminal encoding region of a partial-length *L. hesperus MiSp* cDNA (HM752571; primers listed in Additional file 1: Table S3). An ~49 kb *MiSp* containing clone was shotgun sequenced and assembled to 8× coverage by Qiagen (Hilden, Germany).

#### PCR amplification and cloning of *Latrodectus* genomic *MiSp*

We amplified full-length *L. hesperus MiSp* from genomic DNA using primers designed from the 3’ UTR of the C-terminal encoding *MiSp* cDNA (HM752571) and the N-terminal region of another partial *MiSp* cDNA (HM752570). We amplified genomic *MiSp* from *L. tredecimguttatus* and *L. geometricus* first using primers designed from *L. hesperus* MiSp N and C-terminal cDNAs and then with internal species-specific primers (primers in Additional file 1: Table S3). Genomic PCR was performed with 1 unit of AccuPrime^TM^ *Taq* DNA Polymerase High Fidelity (Invitrogen, Carlsbad, CA) according to manufacturer’s instructions. Cycling conditions were 40 cycles of 94 **°**C for 30 s, 50–60 **°**C (depending on primer pair) for 45 s, and 68 **°**C for 10 min.

PCR products were gel-excised and cloned using the TOPO®-TA Cloning® Kit (Invitrogen). Clones were screened by PCR amplification of MiSp N and/or C-terminal encoding region, and *MiSp* positive clones were sequenced with Sp6, T7, M13F, or M13R universal primers. End sequencing of TOPO clones indicated that, within both *L. geometricus* and *L. tredecimguttatus*, clones clustered into two distinct groups (Additional file 1: Figure S2). For both *L. tredecimguttatus* and *L. geometricus*, the longest clones representing each cluster were completely sequenced. End sequences and restriction digest patterns of *L. hesperus* TOPO clones were very similar. Thus, one clone was chosen for complete sequencing. Clones chosen for complete sequencing were subjected to random transposon insertion with the GPS-1 Genome Priming System (New England Biolabs, Ipswich, MA). Position of transposon insertion was mapped with restriction digests and clones were sequenced from both ends of the transposon. Sequences were assembled with Sequencher v. 4.9 (GeneCodes).

#### *Steatoda grossa* and *P. tepidariorum* cDNA libraries

We constructed cDNA libraries from *S. grossa* total silk glands and *P. tepidariorum* major and minor ampullate silk glands following procedures detailed in Garb et al. (2010). In brief, total RNA was extracted with TRIzol® (Invitrogen) and the RNeasy Mini Kit (Qiagen, Valencia, CA). mRNA was isolated with oligo-(dT)-tagged magnetic beads (Invitrogen). cDNA was synthesized with SuperScript®III (Invitrogen), fractionated by size with ChromaSpin 1000 columns (Clontech, Mountain View, CA), blunt-end ligated into pZErO^TM^-2 (Invitrogen), and electroporated into TOP10 *Escherichia coli* cells (Invitrogen). Approximately 2000 colonies per library were arrayed and stored at −80 °C. The libraries were screened by gel electrophoresis of plasmid DNA [50]. Plasmids with inserts >500 bp were sequenced with T7 or SP6 universal primers. We identified silk protein-encoding sequences by conceptual translations and comparisons to published sequences with BLASTX [51].

An *S. grossa* cDNA clone (designated *Sg* MiSp) with a 3 kb insert containing the C-terminal encoding portion of MiSp was completely sequenced by random insertion of transposons with the EZ-Tn5 < Tet > kit (Epicentre). Transposon mapping, sequencing, and assembly were performed as for TOPO clones above.

#### Amplification and cloning of *S. grossa* and *P. tepidariorum MiSp*

We attempted to amplify complete MiSp encoding sequences from *S. grossa* genomic DNA using primers designed from conserved regions of *Latrodectus* MiSp N-terminal encoding sequences and from the C-terminal encoding region of the *S. grossa MiSp* cDNA (Additional file 1: Table S3). *S. grossa* genomic DNA was amplified with 1 unit of Phusion® High-Fidelity DNA Polymerase (New England Biolabs), 200 μM each dNTP, 0.5 μM each primer, 1X Phusion® GC buffer, and 3% DMSO. Cycling conditions were 40 cycles of 98 **°**C for 5 s, 58 **°**C for 15 s, and 72 **°**C for 2.5 min. PCR products were gel-excised and cloned using the TOPO®-Blunt Cloning® Kit (Invitrogen). End sequencing of these clones revealed numerous premature stop codons and thus clones were not completely sequenced.

In order to obtain longer N-terminal MiSp encoding sequences from *S. grossa*, we amplified *S. grossa* genomic DNA using forward primers designed from the conserved region of *Latrodectus* MiSp N-terminal encoding sequences or from the *S. grossa* N-terminal *MiSp* cDNA, and a reverse primer designed from the repetitive region of the *S. grossa* C-terminal cDNA (Additional file 1: Table S3). Genomic DNA was amplified with 1 unit GoTaq Polymerase (Promega), 1X GoTaq Buffer, 200 μM each dNTP, 0.5 μM each primer, and 1.5 mM MgCl_2_ with 45 cycles of 30s at 94 °C, 45 s at 54.2 °C or 61 °C, and 1.5 or 2 min at 72 °C. PCR products from the two amplifications were separately gel-excised and cloned using the TOPO®-TA XL PCR Cloning^**®**^ Kit (Invitrogen). TOPO clones from each reaction were amplified with M13 forward and reverse primers and sequenced with T7 or SP6 universal primers.

A *MiSp* gene was identified in the *P. tepidariorum* genome by BLASTN searches of our N- and C-terminal encoding MiSp cDNA sequences against the genome assembly [52] generated by the i5K initiative [53]. We found alignments of both cDNAs to a region on Scaffold 853 that included an Augustus-predicted gene model (aug3.g7951.t3). Using the genomic sequence, we designed a reverse primer in the C-terminal encoding region and a forward primer in the adjacent repetitive region. Genomic DNA and cDNA were amplified as for *S. grossa* N-terminal encoding region but with 40 cycles of 30s at 94 °C, 45 s at 60.1 °C and 1 min at 72 °C. PCR products were gel-excised and sequenced. Genomic PCR products had sizes ranging from 250 bp to 2500 bp, while cDNA PCR products had sizes ranging from 250 bp to 1200 bp. Those bands that were too large to directly sequence were TOPO cloned using the TOPO-TA PCR Cloning Kit (Invitrogen) and completely sequenced by random insertion of transposons as for *S. grossa* TOPO clones above. Completed TOPO clones were compared to the *P. tepidariorum* genome by BLASTN.

### Amino acid sequence characteristics

Amino acid composition and codon usage were calculated with codonW [54]. Kyte-Doolittle hydropathy plots [55] with a window size of seven were generated online [56]. Amino acid motifs GGX, A_n_ (*n* ≥ 4), (GA)_n_ (*n* ≥ 2), and GPG were identified by word searches in Microsoft Word. Spacer regions were defined manually as regions that were not in the N or C-termini and were low in alanine and glycine and were manually aligned. Two authors (JVH and CYH) independently assigned spacer regions. Tandem repeats were identified by XSTREAM v1.4.8 using default parameters for moderate repeat degeneracy and high significance [57].

### Transcript abundance estimation

Transcript abundance was estimated by aligning raw RNA-seq reads [58] (SRR1539569 and SRR1539569 for *L. hesperus* and *L. geometricus* respectively) to our full or almost full-length *MiSp* paralogs found in *L. hesperus* (*Lh MiSp_v1 and MiSp_v2*) and *L. geometricus* (*Lg MiSp_v1 and MiSp_v2*) using Bowtie 2 with default parameters, which searches for multiple alignments for each read but reports the one best alignment [59]. RNA-seq reads were generated for mRNA isolated from major and minor ampullate glands of ~30 adult females and from all the silk glands (“total silk”) of a single adult female for both *L. hesperus* and *L. geometricus*. SAMtools v1.1 was used to report the number of sequence reads matching our reference *MiSp* sequences [60]. To compare transcript abundance between loci within the same library, we first compared the total counts of reads aligned to the entire reference sequence. We did not correct for gene length because many of our libraries were heavily 3’-prime biased. However, to ensure that differences in gene length did not dramatically affect our estimates of the ratio of one MiSp locus to another, we also compared the counts that aligned to the last 500 bases of coding sequence (Table 2).

### Relationships among spidroin paralogs

We added our theridiid and *A. ventricosus* [30] MiSp N and C-terminal coding sequences to an alignment of 29 spidroin termini analyzed previously (see [17]). N-terminal sequences were not available for many spidroins and thus we added 25 additional spidroin sequences to the C-terminal alignment to more comprehensively represent spidroin gene family diversity in our analyses (Table 1). Additional C-termini of spidroin paralogs were chosen for species for which MiSp has been characterized. The expanded amino acid alignment was manually edited and used to guide the nucleotide alignment in SeaView v. 4.2.7 [61, 62].

We conducted heuristic searches for maximum parsimony (MP) and maximum likelihood (ML) trees based on amino acid and nucleotide alignments in PAUP* v4.0b10 using tree bisection reconnection branch swapping and 1000 (MP) or 10 (ML) replicates of random stepwise addition of taxa [63]. Support for clades recovered in MP analyses was evaluated with 1000 bootstrap pseudoreplicates and 10 random addition sequences per pseudoreplicate.

Bayesian analyses were carried out with MRBAYES v.3.1.2 [64, 65]. Optimal models of evolution were determined for nucleotide sequences with JMODELTEST v0.1.1 [66] and for protein sequences with PROTTEST v2.4 [67] for N and C-termini separately. Combined analysis of nucleotides employed a model partitioned by N and C-termini. Combined analysis of amino acids employed a mixed model, which allowed estimation of the optimal model of protein evolution during the Bayesian analysis. Default priors and Metropolis coupled, Markov-chain, Monte Carlo sampling procedures were executed for two independent runs of 10 million generations each, sampled every 1000th generation. Convergence was assessed every 1000th generation and the posterior distribution was considered adequately sampled if the standard deviation of split frequencies of these two runs was below 0.01. The mygalomorph spidroin, *Bothriocyrtum californicum* fibroin1 (*Bc* fibroin1), was set as the outgroup in all analyses.

### Gene tree – species tree reconciliation

We inferred gene duplication events by reconciling each of our gene trees with two species trees (Fig. 1) using Notung v. 2.8.1.7 [68]. Species trees were based on published phylogenies as follows. Relationships among families were based on Garrison et al. [38] and Dimitrov et al. [39]. These studies agreed on all family-level relationships except the placement of Deinopidae and Uloboridae. The former placed Deinopidae sister to the RTA-clade (represented here by *E. australis* and *A. aperta*) with Uloboridae sister to Deinopidae plus the RTA-clade. The latter switched the placement of Uloboridae and Deinopidae. For relationships among theridiid species we used Liu et al. [35] and Garb et al. [34]. Relationships among nephilid species were based on Kuntner et al. [69]. Relationships among araneid genera were based on Dimitrov et al. [39] with relationships for *Argiope* species from Cheng and Kuntner [70].

### Mechanical properties of minor ampullate fibers

We obtained minor ampullate silk from *L. hesperus*, *L. geometricus*, and *S. grossa* adult females within one week of being received or collected. Individual spiders were anesthetized with CO_2_ for 2–5 min. We then secured the spider ventral side up to a stereo microscope stage using Scotch^**®**^ tape, exposing the spinnerets. We manually pulled silk emanating from the minor ampullate spigots. Single fibers were taped to m-shaped cardstock (2.54 × 7.62 cm), with a single silk fiber suspended across each of the two rectangular notches (1 cm wide × 1.5 cm deep) in a collection card. Fibers were secured onto the cards using cyanoacrylate glue. We collected four to ten minor ampullate fibers from each of seven (*L. hesperus* and *S. grossa*) or eight (*L. geometricus*) individuals per species.

We measured silk fiber diameters using polarized light microscopy (as described in [6]). Tensile tests were conducted with a NanoBionix tensile tester (formerly MTS Systems Corp., Oak Ridge, TN; currently Keysight Technologies, Santa Rosa, CA) [6]. In brief, each m-shaped card was cut in half, such that only one fiber segment was tested at one time. Each silk sample was mounted onto the NanoBionix and extended at a rate of 1% per second until failure. We defined strength as the true stress of the fiber at breakage. True stress is the force applied to the fiber divided by its cross-sectional area calculated from the original cross-sectional area assuming constant volume of the fiber during extension [71]. We defined extensibility as the true strain at breakage. True strain is the natural log of the ratio of the instantaneous length of the fiber to the original gage length of the fiber. We also measured stiffness or Young’s modulus, the initial slope of the true stress-true strain curve, and toughness, the area under the true stress-true strain curve. Force-extension data were visualized using Testworks 4.0 software (MTS Systems Corp.). We tested for differences among species for the four tensile properties using multivariate analysis of variance (MANOVA). We used Tukey’s test for post-hoc pairwise comparisons. All statistical tests were carried out with SPSS 13.0 (SPSS Inc, Chicago, IL).

We compiled published tensile data for minor ampullate silks and fit the relationship between proportion of GPG amino acid motifs and extensibility to linear models implemented in R for each species for which at least one MiSp sequence and minor ampullate fiber tensile data were available. Minor ampullate silk was assumed to be composed only of MiSp and for each species all available MiSp motif percentages were averaged to estimate composition. To account for phylogenetic relationships and evolutionary distances among species we calculated phylogenetic independent contrasts using APE [72] implemented in R. We used estimates of divergence time from three published molecular clock studies [35, 38, 39] for branch lengths (see Additional file 1: Figure S6).

## Additional file

Additional file 1: Contains four supplementary tables and eight supplementary figures. (PDF 1474 kb)
