# Supplementary material for: Duplication and concerted evolution of MiSp-encoding genes underlie the material properties of minor ampullate silks of cobweb weaving spiders
Source: BMC Evol Biol. 2017 Mar 14;17:78. doi: 10.1186/s12862-017-0927-x (PMC5348893; doi:10.1186/s12862-017-0927-x)
Supplement: Additional file 1: — Contains four supplementary tables and eight supplementary figures. (PDF 1474 kb) [file 12862_2017_927_MOESM1_ESM.pdf]

Supplementary Tables

Table S1. Amino acid proportions and codon usage of alanine and glycine in MiSp from *L. hesperus* (*Lh*), *L. tredecimguttatus* (*Lt*), *L. geometricus* (*Lg*), *S. grossa* (*Sg*), and *P. tepidariorum* (*Pt*).

		Lh MiSp_v1	*Lh* MiSp_v2	*Lt* MiSp_v1	*Lt* MiSp_v2	*Lg* MiSp_v1	*Lg* MiSp_v2	*Sg* MiSp[a]	*Pt* MiSp_v1[b]
**% aa**	Alanine	27	30	30	26	22	28	17	27
% Alanine codon	GCU	49	44	42	44	31	46	25	37
	GCA	43	44	44	47	54	44	70	52
	GCC	5	7	6	2	7	4	2	9
	GCG	3	6	7	8	8	6	3	2
**% aa**	Glycine	34	36	35	31	30	32	31	38
% Glycine codon	GGU	40	38	39	39	42	31	25	38
	GGA	45	49	47	38	42	48	67	49
	GGC	12	12	11	19	10	14	6	11
	GGG	3	2	3	4	6	3	3	2

See Table 1 for *Latrodectus* accession numbers.

[a] Approximately 3 kb partial C-terminal encoding cDNA, KX584021.

[b] Based on combining the incompletely assembled MiSp-encoding region from Scaffold 853 of the i5K genome with an ~2.3 kb TOPO-cloned PCR product, KX584004.

Table S2. Tandem repeats identified by XSTREAM from selected MiSp variants for *L. hesperus* (*Lh*), *L. tredecimguttatus* (*Lt*), *L. geometricus* (*Lg*), *S. grossa* (*Sg*), and *P. tepidariorum* (*Pt*). *Sg* MiSp is based on an approximately 3 kb partial C-terminal encoding cDNA, KX584021. Accessions for partial length *Sg* MiSp N-terminal sequences are in Table S4. *Pt* MiSp_v1 is based on combining the incompletely assembled MiSp-encoding region from Scaffold 853 of the i5K genome with an ~2.3 kb TOPO-cloned PCR product, KX584004. Amino acid motifs are color coded as follows: A_n = red, GA_n = purple, GGX = green, GPG = blue, spacers = bold.

identifier	start	end	period	copy#	consensus error	consensus (no gaps)
Lh MiSp_v1	219	1107	424	2.1	0.13	SQAAGSAAAGGYGQGSAGSYGNAAAGAAGAGAGGYGQGAGAAAGAGAGGYGQGAGSYGQGAGAAAGGAGAGAGGYGQGAGGYGQGAGAAAGAAGGAGSGGYGLGAGIGAGAAAIAGGYGQGAGAAAGAAAGAGAGEYGQGAGGYGQGAGAAAGAAAGAGAGGYGQGAGGYGQGAGAAGQGAGAAAGAGAGGYGQGAGGYGQGAGAAAGAAAGAGAGGYGQGAGGYGQGAGAAAGAAAGAGAGGYGQGAGGYGQGAGAAAGAGAGGYGRGAGSAAGAAAGSGAGGYGQGAGGYGQGAGAGAGGYGQGAGASTGAAAGAGAGGYGQGAGGYGQGSGAAAGAGGYGQGSQGYEQGAAATSSAAAGA**SSTGYTERQNEVVTTVTTTRQEIADRRQ**AASASGAVSTSAAAGYGQGAGTGAGGYGQGAGG
Lh MiSp_v1	1812	1940	68	2	0.18	AGAGAYGQGALFGYAQGAGGYGQAAGAAAGAGAGGYGQGAGAGAAAGAAAGTGAGGYGQGAGVGAAAG
Lh MiSp_v1	1156	1306	47	3.17	0.18	AGGYGQGAGAGAGAAAGAIAGGYGQGAGGYGQRGGAAAGAAAGAGAG
Lh MiSp_v1	1458	1505	26	2	0.18	GGGYGRGSAGGAGAGAGAASGAAAGA
Lh MiSp_v1	1032	1240	22	8.91	0.19	GAGGYGQGAGGYGQGAGAAAGA
Lh MiSp_v1	1499	1553	21	2.62	0.16	AAAGAGGYAQAAGGYGQGASV
Lh MiSp_v1	1679	1716	18	2	0.15	AGSGAGGYGQGTGGYGQG
Lh MiSp_v1	1523	1629	16	6.5	0.18	AAAGAGGYGQGAGAAG
Lh MiSp_v1	1410	1439	15	2	0.13	GGYGQGAGAAAGAGA
Lh MiSp_v1	1441	1460	10	2	0.09	AGATGRYGQG
Lh MiSp_v1	172	185	7	2	0.14	GGYGQGS
Lh MiSp_v1	1653	1676	7	3.43	0.08	GAGGYGQ
Lh MiSp_v1	1613	1649	7	5.29	0.14	GAGGYGQ
Lh MiSp_v1	1404	1417	7	2	0.14	GYGKGAG
Lh MiSp_v2	812	1622	28	27.54	0.19	GAGGYGQGAGGYGQGQGAGAAAGAAAGA
Lh MiSp_v2	220	652	26	16.65	0.18	AAAGAAAGAGAGGYGQGAGGYGQGAG
Lh MiSp_v2	149	162	7	2	0.14	GGYGQGS
Lh MiSp_v2	1645	1658	7	2	0.14	GYGQGAG
Lh MiSp_v2	1630	1643	6	2.33	0.07	GAGAAA

Table S2 Continued						
identifier	start	end	period	copy#	consensus error	consensus (no gaps)
Lt MiSp_v2	812	1988	569	2.07	0.03	AAAGYGQGTKGYGQGPGAAAGSGGYGQGAGGYGQGAAVGSAAGSEVAGYRQGAGGYGQGVGAAASAAAGAGAGGYGQGAGGYVQGAGFAAGAAAGAGAGGYGQGAGGYGQGAGSAAGAAAAAAGAGGYGQDAGRYTQGAGFAAGAAAGAAAGGYGKDAGAAAGAGTGAGGYGQGSGGYGQNAGAAAGSGANGQGAGGYGQGAAAVAAAGAGAGGYGQGAGGYGQDAGGYGQGAGGNGQGVVDAAGYGPGSQGYGQSAAATSSAAAGA**SATGYTERQNEVVTTVTTTRQETADRRQ**AARASAAVSTSAAAGYGQGTRGYGQVPGAAAGAGGYGQGAGGYGQGAAVGSSAGSGVAGYGQGSGGYGQGAAAAAGAGAYGQGAGGYGQGAGAATGSGAGGCGQGAGGYGQDAGAAAGAYGQGAGGYGQGAASGVATGTGAGGYGQGAGGYGQGASATAVAAAGAGAGIIGQGAGVYGQGAVSAAGAAGDTGAGGYGQSTGGYGPGFGAGAGAAAGAGGYGPGSQGYGQGAASTSSAAAGA**SSTGYTERQNEVVTTVTSTRQETADRRQ**AARAS
Lt MiSp_v2	262	807	246	2.22	0.14	GGYGQGVAAVAGAAAGAEGYGPGSQGYGQGAAATSSAAAGA**SSTGYTERQNEVVTTVTTTRQETADRRQ**AARASAAAGYGPGAGGNGQGPSAAAGAGGYGQGAGGYGQGAGSAAGAAAGAGAGGYGQDNGAAAGAGQGARGAAAGSGGYGQGAGTAAGAAAGAGAGGNGQGAGGYALGAGAAAGGAAGAGAGGYGQGAGGYGQGAGFAAGVSAAGAGAGGYGQGAGGAAQGAGGYGQGAGGYGQGA
Lt MiSp_v2	173	252	26	3.08	0.08	AGGYGQGAGAAAGAGAGAGAGGYGQG
Lt MiSp_v2	226	282	17	3.24	0.14	GGYGQGAGAAAGAAAGA
Lt MiSp_v2	2040	2055	8	2	0.19	SSSSASGT
Lt MiSp_v2	1990	2003	7	2	0.14	GYGQGRG
Lt MiSp_v2	2094	2105	7	2	0.2	SAAVSRL
Lt MiSp_v1	324	571	25	9.48	0.18	AGGYGQGAGAAAAGAGAGAGGYGQG
Lt MiSp_v1	1095	1319	24	9.21	0.19	GAGAAAGAGAGGYGQGAGGYGAGA
Lt MiSp_v1	644	1070	24	17.04	0.2	GAGAGGYGQGAGGYGAGQGAAAAA
Lt MiSp_v1	216	251	18	2	0.08	AAAGAAGAGAGGYGQGAG
Lt MiSp_v1	1349	1364	8	2	0.19	SSSSASGT
Lt MiSp_v1	145	158	7	2	0.14	GGYGQGS
Lt MiSp_v1	634	655	7	2.86	0.14	YGQGAGG
Lt MiSp_v1	569	582	7	2	0.14	GYGQGAA
Lt MiSp_v1	1403	1414	7	2	0.2	SAAVSRL

**Table S2 Continued**						
identifier	start	end	period	copy#	consensus error	consensus (no gaps)
Lg MiSp_v1	186	784	290	2.07	0.12	GIGGYGLGAGGYGQAAAATAGATAGAGGYGQGADGVSGAGPRGAPAGYGPGAGPAAGADSGAVAGGKGPGGYGPSQGASASSSAAAAAASA**GYTQKQNEVITTVSTTRQEIADYGQKQ**ASGASAAVSTSSAGGYAQGPGGYGPGKGAGATAGAGAGGYSQGPGGYAQGVSTAAGAVAIAGAGGYGPGSTGPYGQGAIDASGGYGPGAGTAAGASASAGAGAATGVGPGGYGQGLGGYGQAAGPGAGGYGQGAGAAAGAGTAAGIGGYGPGAGGFGQGVGAAAGAATDAGP
Lg MiSp_v1	1048	1570	266	2	0.05	GIYGQGAGGYRQGVGAAAGAATGVTAGAGGYDQAAGVSGAGLRGAPGGYGPGAGISAGAASGAVAGGKGPGGYGPSQVATASSSAAAAAAAS**GYTQRQNEVITTVSTTRQKTADYGQKQ**ASGASAAVSSSSAGGYTQGPGGYGPGQGAVAGGYGPGAGSYGAGAIDASGGYGQGAGTAAGASASAGAGAATGVGPGGYGQGLGGYGQAAGQGAGGYGQGAGTAAGASASAGAGAATGVGPGGYGQGLGGYGQAAGAAIGATAGTGA
Lg MiSp_v1	903	917	7	2.14	0.18	GGYAQGP
Lg MiSp_v1	998	1012	7	2.14	0.13	GGYGQAA
Lg MiSp_v1	1586	1606	7	3	0.14	GGYGQAA
Lg MiSp_v1	1758	1767	5	2	0.1	SAGAG
Lg MiSp_v2	218	281	31	2.06	0.1	YGQGAGAAGAGAAAGAGAGGYGQGAGAGAGG
Lg MiSp_v2	281	714	26	16.38	0.18	GGYGQGAGAGAGAAAGAGAGGYGQGA
Lg MiSp_v2	698	726	9	3.11	0.16	AGGYGRGAG
Lg MiSp_v2	149	162	7	2	0.14	GGYGQGS
Lg MiSp_v2	814	834	7	2.71	0.14	SSAASRI
Sg MiSp	3	404	164	2.45	0.13	AAAASAPGASGYGPGVSGYGPGQGAGPGQGAGPGQGVGPGQGAGTGGQGSGASSAAAASAGTSGYGPGVSGYGPGQGAGPGGQGSGASSRAAATSAGTRGYGPGYPGYGQGPASGPTAADKYGPGIGGYAPG**RSTTSTSAATASATTVDIGPQI**GGYGPGQGIG
Sg MiSp	345	552	59	3.42	0.1	GPGQGAGPGGQGSGASSAAAASAGTSGYGPGVSGYGPGQGAGPGQGAGPGQGAGPGQGV
Sg MiSp	614	693	38	2	0.11	AAAAGASGYGPGVSGYGPGQGAGPGQGAGPGQGTGLGA
Sg MiSp	928	945	8	2.25	0.16	SASALSSP
Sg MiSp	715	730	7	2	0.19	SGYGPGA
Sg MiSp	830	843	7	2	0.07	SGYGPGQ

**Table S2 Continued**						
identifier	start	end	period	copy#	consensus error	consensus (no gaps)
Sg A13	287	333	6	7.5	0.15	GYGPGQ
Sg A1	338	351	7	2	0.07	SGYGPGQ
Sg A1	286	311	6	4.17	0.19	GYGPGQ
Sg B5	152	209	26	2.23	0.09	GYGPGQDIGAAASPTSGASGYGPGAS
Sg B5	206	240	6	5.5	0.17	GPGQGA
Sg B5	299	337	6	6.17	0.13	GAGPGQ
Sg B5	133	143	5	2.2	0.08	TTSAT
Pt MiSp_v1	1127	1341	81	2.63	0.15	GVGAGAGAGAGAAAGYGAGAGAGAGAGAGAAAGAGGYGQGAGQGYGAGAGAGAGAAGGTGQGTGQGYGAGAGSGAGAEAAA
Pt MiSp_v1	163	533	30	10.77	0.17	GGAGGYGQGAGQGYGAGAGSGAGAGAGAGA
Pt MiSp_v1	518	1126	28	20.46	0.19	GAGQGYGAGAGSGAAAGAGAGAAGGYGQ
Pt MiSp_v1	1392	1441	21	2.38	0.06	AGAGGYGQGAGQGYGAGAGAG

**Table S3.** Primers shown 5' to 3'.	
Primer Name	Primer
Screen *L. hesperus* genomic library for MiSp	
GQAQVSRA_F	AGGGGCTTGGATAACAACTTCAC
SEVVIQAP_R	GGACAAGCACAAGTTTCACGAGC
Checked genomic clone for N-term (designed from HM752570)	
LhMiSp_N_47F (also used with SEVVIQAP_R for initial Lg and Lt TOPO clones)	GCTTCGTCTCTTTAGATGCCGC
LhMiSp_N_517R	CACCACTTTGCTGTCCGTAACC
L. hesperus genomic vs. cDNA specific primers.	
Misp_DwncdnaR (designed from HM752571; used with LhMiSp_N_47F to amplify almost complete MiSp from genomic DNA that matched the original cDNA)	ACTAATTATTTCCTTAAAGATAG
misp_dwngenR (used with GQAQVSRA_F to check that genomic clone could be amplified from genomic DNA)	GTATGTATTTAGCTGAAGATATTC
misp_cF117	TGTTAGTGGTGGAAGTGC
misp_cF291	CAGTCCTGGAATGTCATC
L. geometricus specific primers to TOPO clone	
geoMiSp_N_F	GCTGGAGTATTGTCTCGTTCC
LgMiSp_c-172R	GGAAGCAGCAGAACTCAATCGTG
geo_MiSp_N_R (used with geoMiSp_N_F)	GTGCTGCTGAAGATGTTGACA
L. tredecimguttatus specific primers to TOPO clone	
treMiSp_N5_F	GAGAGAAACCCAAACAACTCG
LtMiSp_c-122R	TCAATACTCCTCCAGTTGCTAGGC
LtMiSp_c-390F (used with LtMiSp_c-122R)	GGCATCCGCATCAGCTAATG
To check that TOPO clone exists in *L. geometricus* genome	
LgM19_cF249 (used with LgMiSp_c-172R or MiSpR)	GGTTCTGGAGCAGGAGGAAAAG
LgM19_cF477 (used with LgMiSp_c-172R or MiSpR)	GGAGGCAGTGGTCCAGTTCAAG
LgM19_nF139 (used with LgM19_nR428 or R464)	CCTTCGCATCTTCAGTTTCGG
LgM19_nR428 (used with LgM19_nF139, LgeoNF, or LhMiSpNF47)	CCAGTTGTGCCTATTGAGGTTCTTG
LgM19_nR464	CAGCAGAAGATGTTTGTGTTGACC
To amplify *S. grossa* genomic	
LhMiSp1NF	ATGCATATTCCAGCTCAGTTATC
Stg93_mispC_R724	TATTTCCAACGGAGCCAAAG
To amplify N-terminus of *S. grossa*	
LhMiSp1NF	above
Stg93MiSpRepR233	GGTAGCTGCTGTTGCAGATG
Stg459_F208	ATGCATTGAAGGGAGCATTC
To amplify C-terminus *S. grossa* (not TOPO cloned)	
Stg93_mispRep_F233	CATCTGCAACAGCAGCTACC
Stg93_mispC_R724	above
MiSp_7951_551F	TGGTGCAGGAGGTTACGG
MiSp_7951_875R	AGCCACCAATTCCAGAAGAG

**Table S4.** Accessions for TOPO cloned sequences	
Identifier	Accession Number
Lg M4	KX584024
Lg M5	KX584005
Lg M6	KX584025
Lg M7	KX584008
Lg M8	KX584009
Lg M9	KX584010
Lg M10	KX584006
Lg M16	KX584007
Lg M19	KX584023
Lg M20	KX584011
Lg M22	KX584012
Lt M3	KX584027
Lt M5	KX584033
Lt M6	KX584029
Lt M9	KX584018
Lt M10	KX584028
Lt M11	KX584017
Lt M12	KX584016
Lt M13	KX584030
Lt M14	KX584031
Lt M16	KX584032
Lt M17	KX584013
Lt M18	KX584014
Lt M19	KX584015
Lt M20	KX584026
Sg A1	KX584035
Sg A2	KX584039
Sg A3	KX584041
Sg A4	KX584036
Sg A13	KX584037
Sg A16	KX584038
Sg A22	KX584040
Sg B5	KX584052
Sg B6	KX584053
Sg B8	KX584054
Sg B10	KX584042
Sg B11	KX584043
Sg B12	KX584044
Sg B23	KX584045
Sg B27	KX584046
Sg B29	KX584047
Sg B31	KX584048
Sg B36	KX584049
Sg B46	KX584050
Sg B47	KX584051

Supplementary Figures

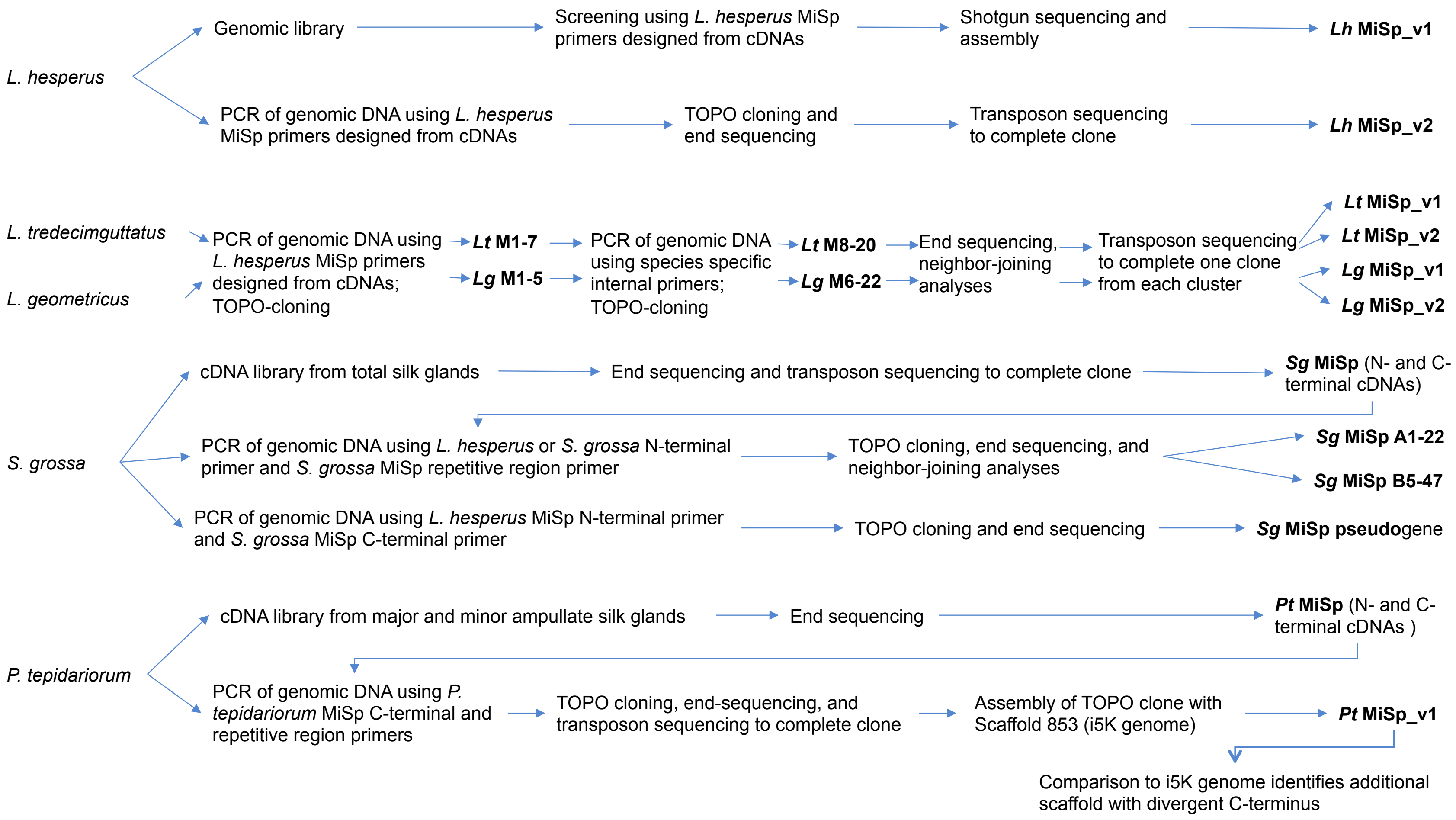

Figure S1. Schematic of molecular methods used to characterize MiSp-encoding loci in five species of cobweb weavers.

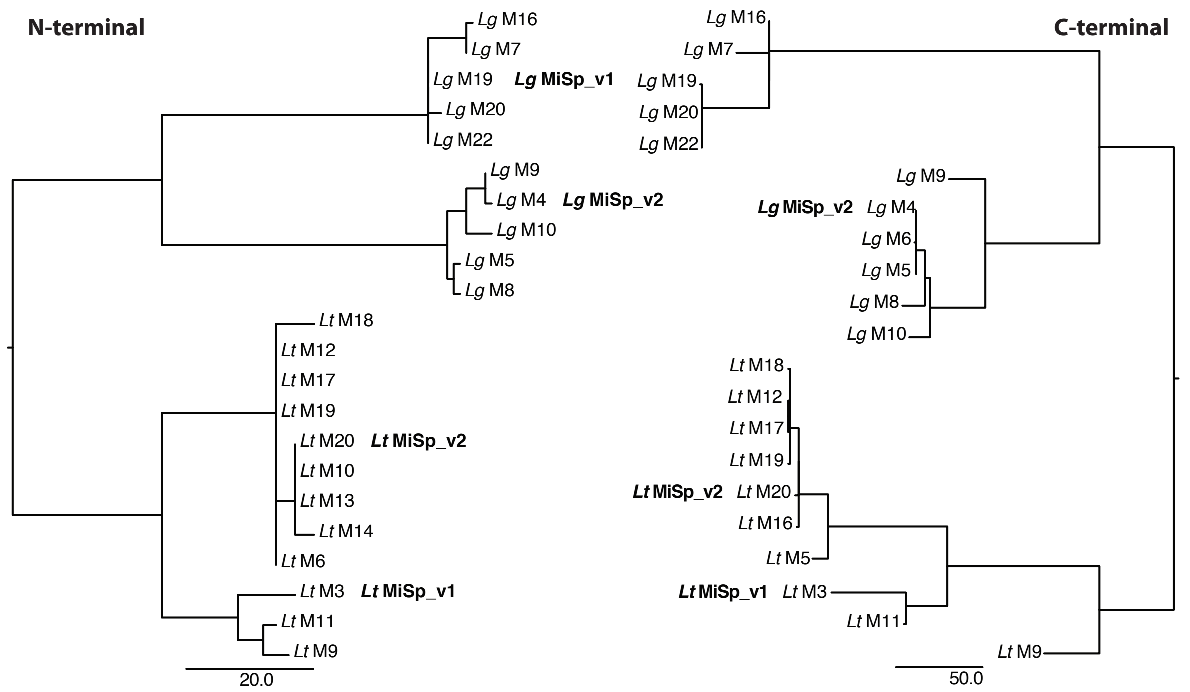

Figure S2. Neighbor joining trees for N-terminal and adjacent repetitive (left) and C-terminal and adjacent repetitive (right) MiSp encoding sequences from TOPO clones for *L. tredecimguttatus* (*Lt*) and *L. geometricus* (*Lg*). Completely sequenced clones indicated by bolded names (see Table 1 and Table S4 for accessions). Trees are midpoint rooted. Units are number of substitutions.

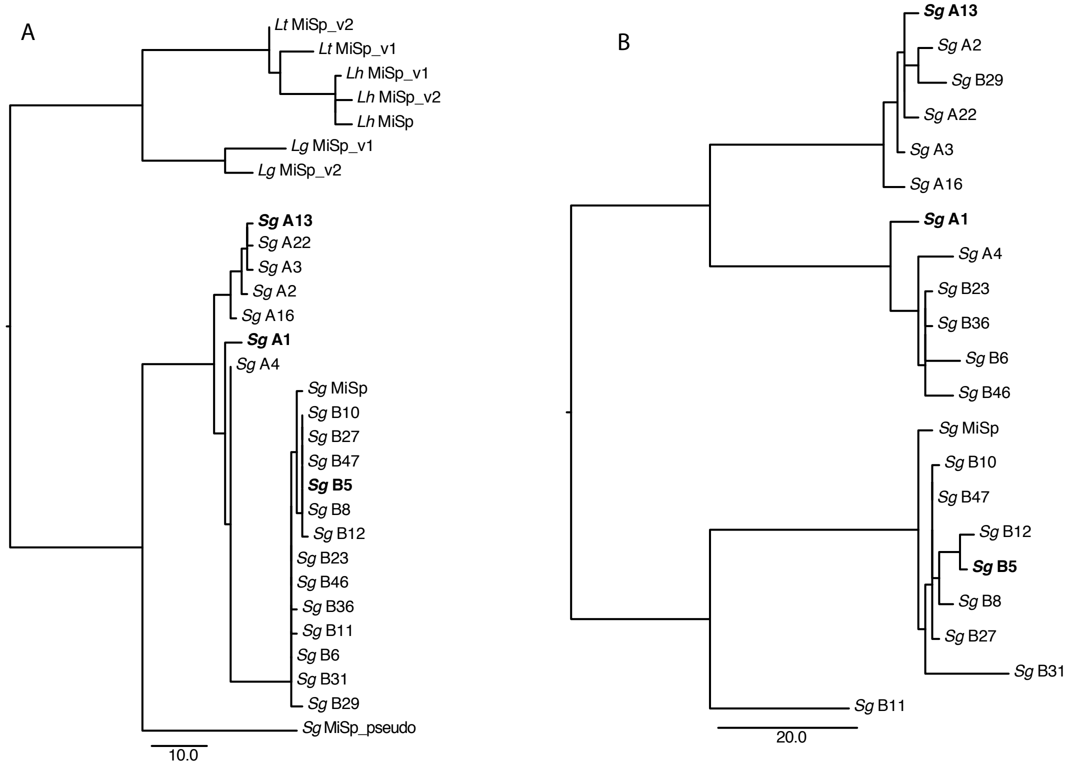

Figure S3. Neighbor joining trees for (A) N-terminal MiSp encoding sequences from *L. hesperus* (*Lh*), *L. tredecimguttatus* (*Lt*), *L. geometricus* (*Lg*), and TOPO clones of *S. grossa* (*Sg*); (B) N-terminal and adjacent repetitive MiSp encoding sequences from TOPO clones for *S. grossa* (Sg). *S. grossa* TOPO clones resulted from two separate PCR reactions, which are indicated here as “A#” and “B#”. See Table S4 for accession numbers. Bolded names were chosen as exemplars for inspection of spacer sequences (Figure 4) and tandem repeats (Table S2). Units are number of substitutions.

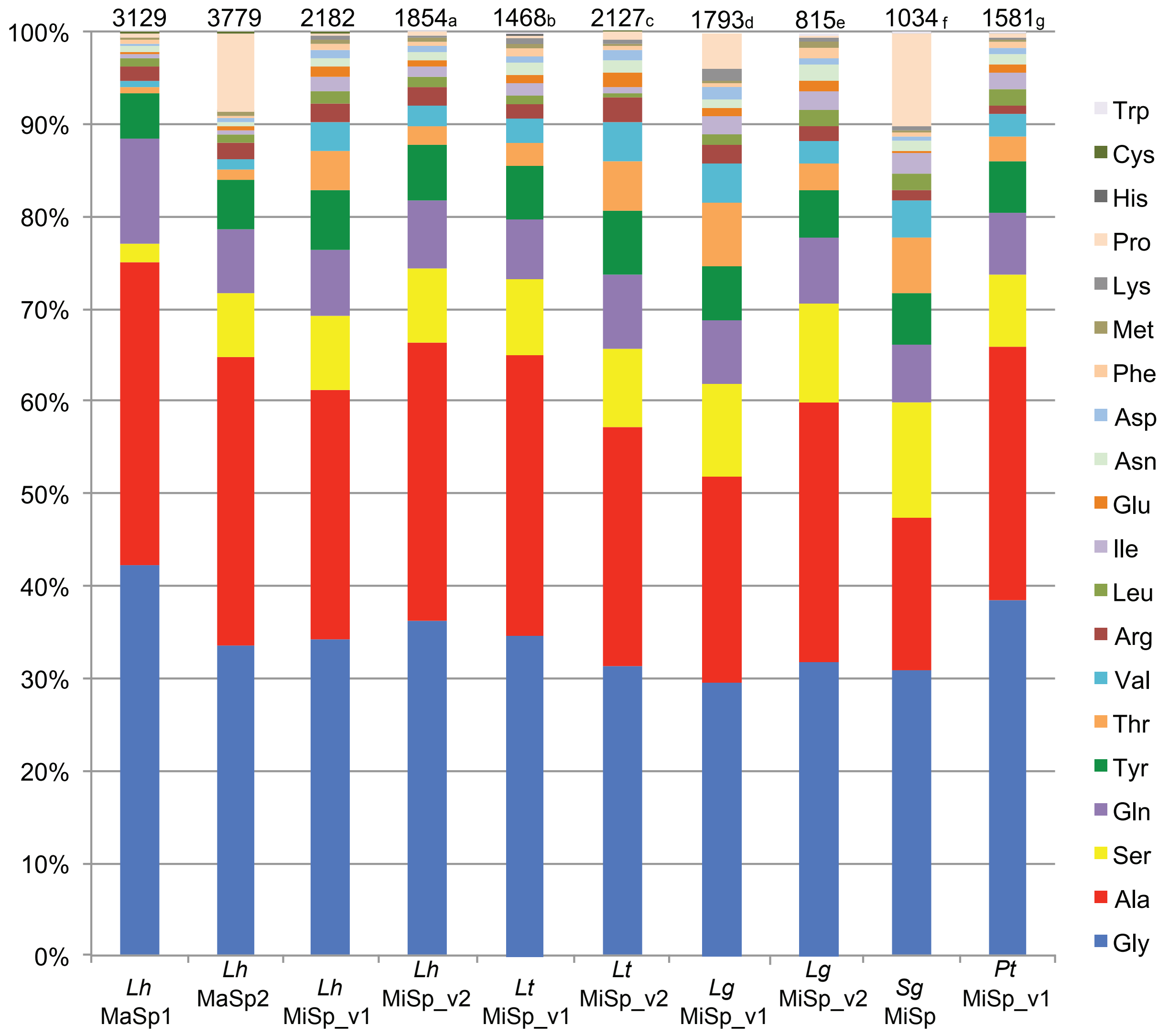

Figure S4. Amino acid proportions for theridiid MaSp1, MaSp2, and MiSp. See Table 1 for *Latrodectus* and *S. grossa* MiSp accession numbers. Proportions are based on full-length *L. hesperus* (*Lh*) MaSp1 (EF595246), MaSp2 (EF595245), and MiSp_v1; almost complete *L. hesperus* MiSp_v2, *L. tredecimguttatus* (*Lt*) MiSp_v1 and MiSp_v2, and *L. geometricus* (*Lg*) MiSp_v1 and MiSp_v2; a partial cDNA for *S. grossa* (*Sg*); and *P. tepidariorum* (*Pt*) MiSp_v1 based on combining the incompletely assembled MiSp-encoding region from Scaffold 853 of the i5K genome with our ~2.3 kb TOPO-cloned PCR product, KX584004. Three letter amino acid abbreviations shown. Lengths of amino acid sequences indicated at top.

[a-e] Based on alignments of N and C-terminal sequences, the estimated length of missing data are as follows: *Lh* MiSp_v2 – 18 aa N-terminus*;* *Lt* MiSp_v1 – 70 aa N-terminus, 80 aa C-terminus; *Lt* MiSp_v2 – 18 aa N-terminus, 40 aa C-terminus; *Lg* MiSp_v1 – 58 aa N-terminus, 100 aa C-terminus; *Lg* MiSp_v2 – 18 aa N-terminus, 40 aa C-terminus.

[f] unknown length of N-terminal plus repetitive sequence missing.

[g] Based on the lengths of gaps reported in Scaffold 853 of the i5k genome, *Pt* MiSp_v1 is missing ~1172 aa of the repetitive region, assuming no introns are present in the gapped regions.

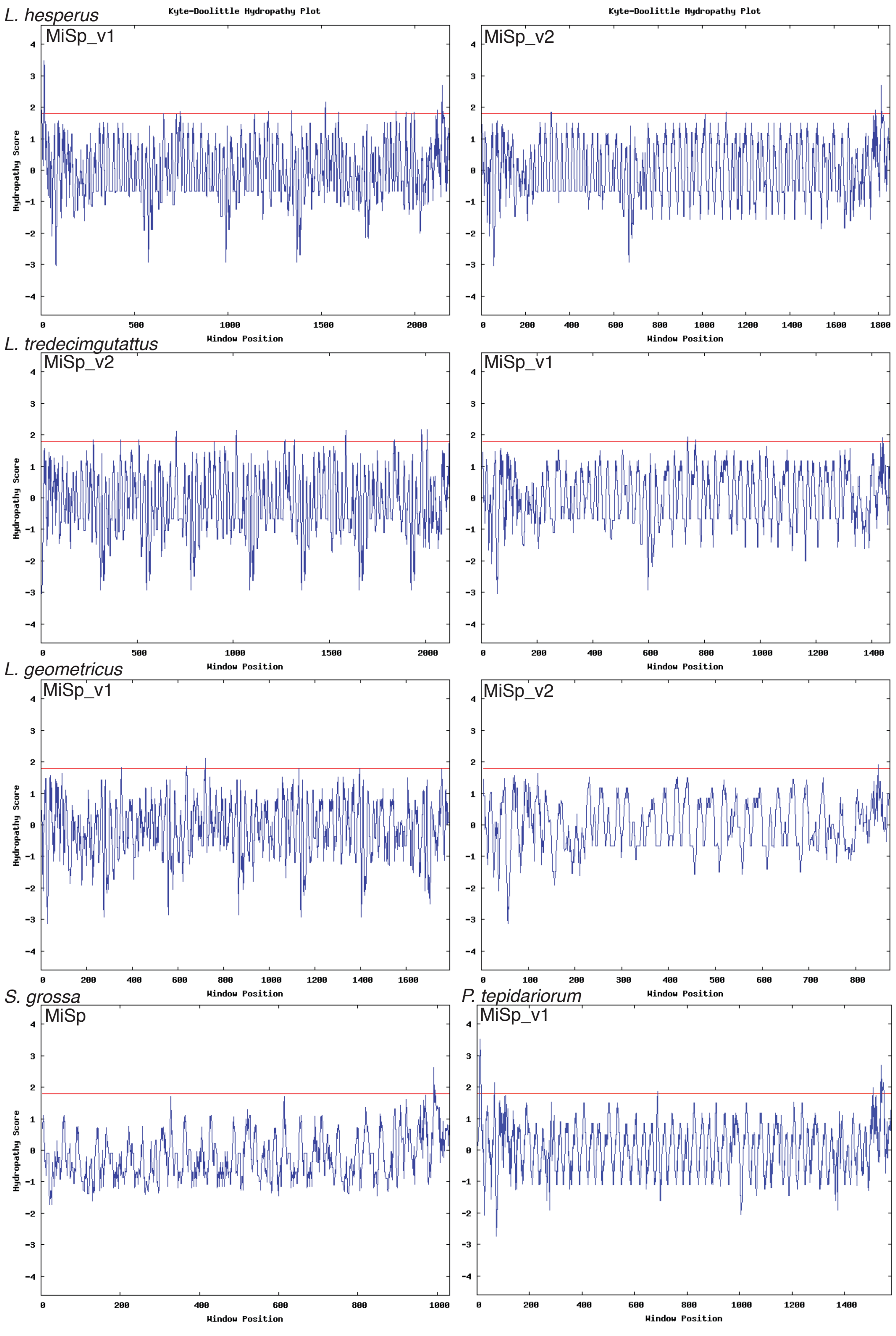

Figure S5. Kyte-Doolittle hydrophobicity plots for cobweb weaver MiSp. *Latrodectus* accessions are in Table 1. *S. grossa* MiSp is based on an approximately 3 kb partial C-terminal encoding cDNA, KX584021. *P. tepidariorum* MiSp_v1 is based on combining the incompletely assembled MiSp-encoding region from Scaffold 853 of the i5K genome with an ~2.3 kb TOPO-cloned PCR product, KX584004.

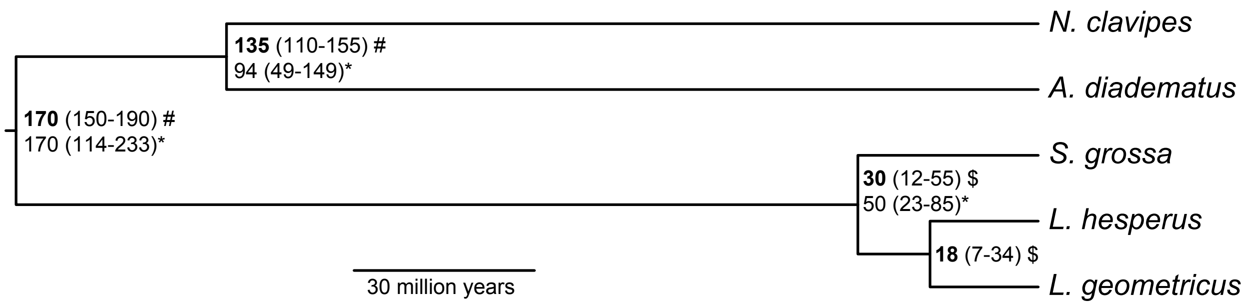

Data for modeling GPG ~ Extensibility	F-statistic	Degrees of Freedom	Adjusted R^2	p-value
Raw	5.44	3	0.53	0.1012
PIC (branch lengths = 1)	9.62	3	0.68	0.0532
PIC (branch lengths = bold)	47.50	3	0.92	0.0063
PIC (branch lengths = youngest)	68.78	3	0.94	0.0037
PIC (branch lengths = oldest)	19.87	3	0.83	0.0210

Figure S6. Top: Phylogenetic relationships among species for which MiSp sequences were available and for which tensile properties of minor ampullate silk fibers had been measured. These data were used to model the linear relationship between the proportion of GPG amino acid motifs in MiSp and the extensibility of minor ampullate silk (bottom). Raw = the averages shown in Table 3 for each species (the average from Liivak et al. 1997 [10] was used for *N. clavipes*). PIC = Phylogenetic Independent Contrasts calculated using various branch lengths. “Branch lengths = bold” refers to the point estimates bolded in the top phylogeny and were considered the best estimates of divergence times. To further evaluate the impact of branch lengths, we also calculated PICs using all branch lengths set to 1 and the youngest and the oldest reported divergence times (shown parenthetically in the phylogeny at top). Divergence times were taken from published molecular clock studies: *=Garrison et al. (2016) [39], #=Dimitrov et al. (2016) [40], $=Liu et al. (2016) [36].

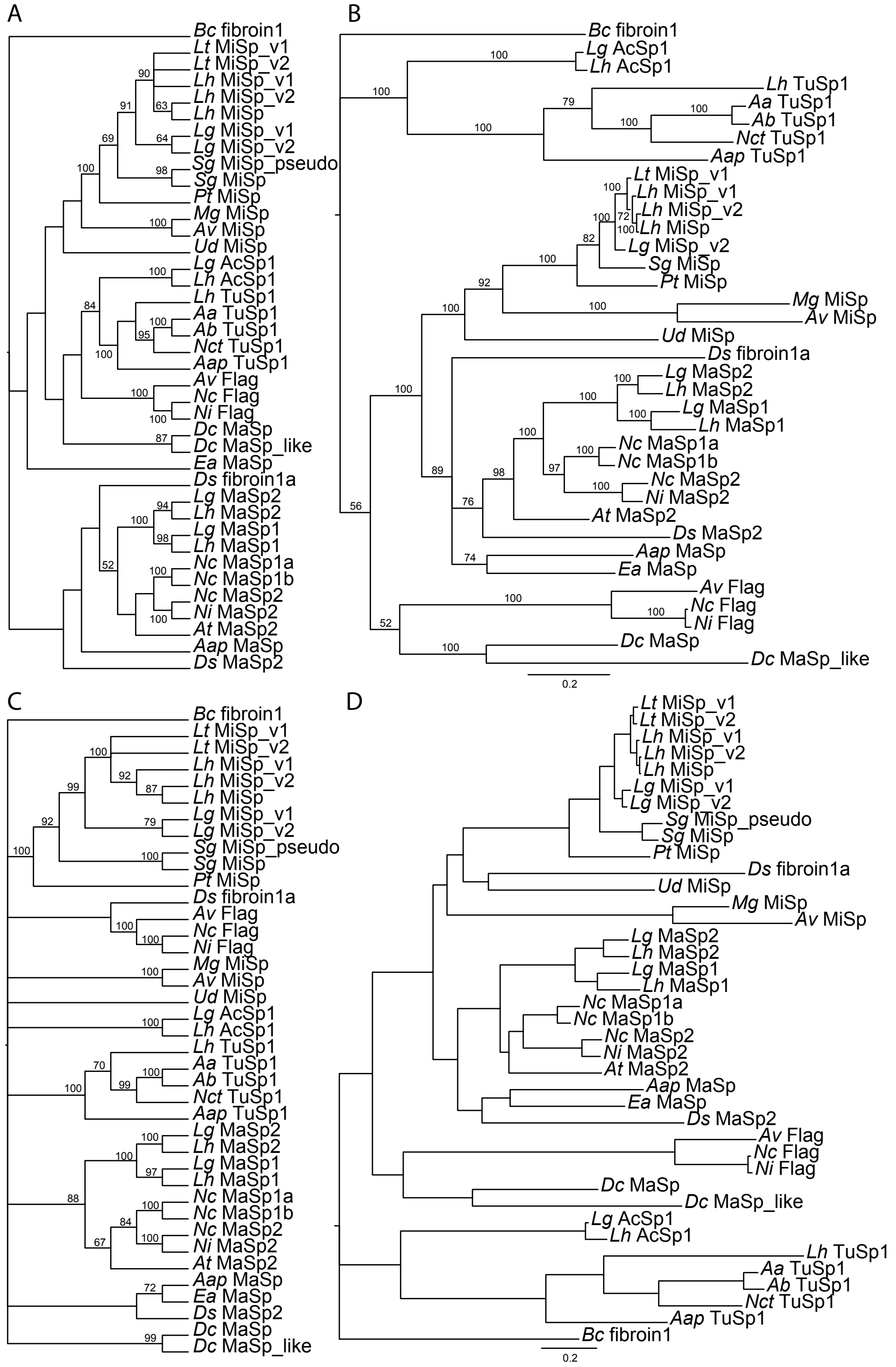

Figure S7. Phylogenetic trees of combined N- and C-terminal spidroin sequences (see Table 1 for accessions). Consensus of maximum parsimony (MP) trees for **A.** amino acid sequence (10 MP trees) and **C.** nucleotide sequence (4 MP trees). Bootstrap values ≥50% are shown above branches. **B.** Bayesian tree for amino acid sequences. Posterior probabilities shown. **D.** Maximum likelihood tree for encoding nucleotides. Units are substitutions per site in B and D. *Sg* MiSp_pseudo was removed from Bayesian analyses of amino acids. *Lg* MiSp_v1 and *Lt* MiSp_v2 were removed from amino acid analyses because their C-termini were < 30aa.

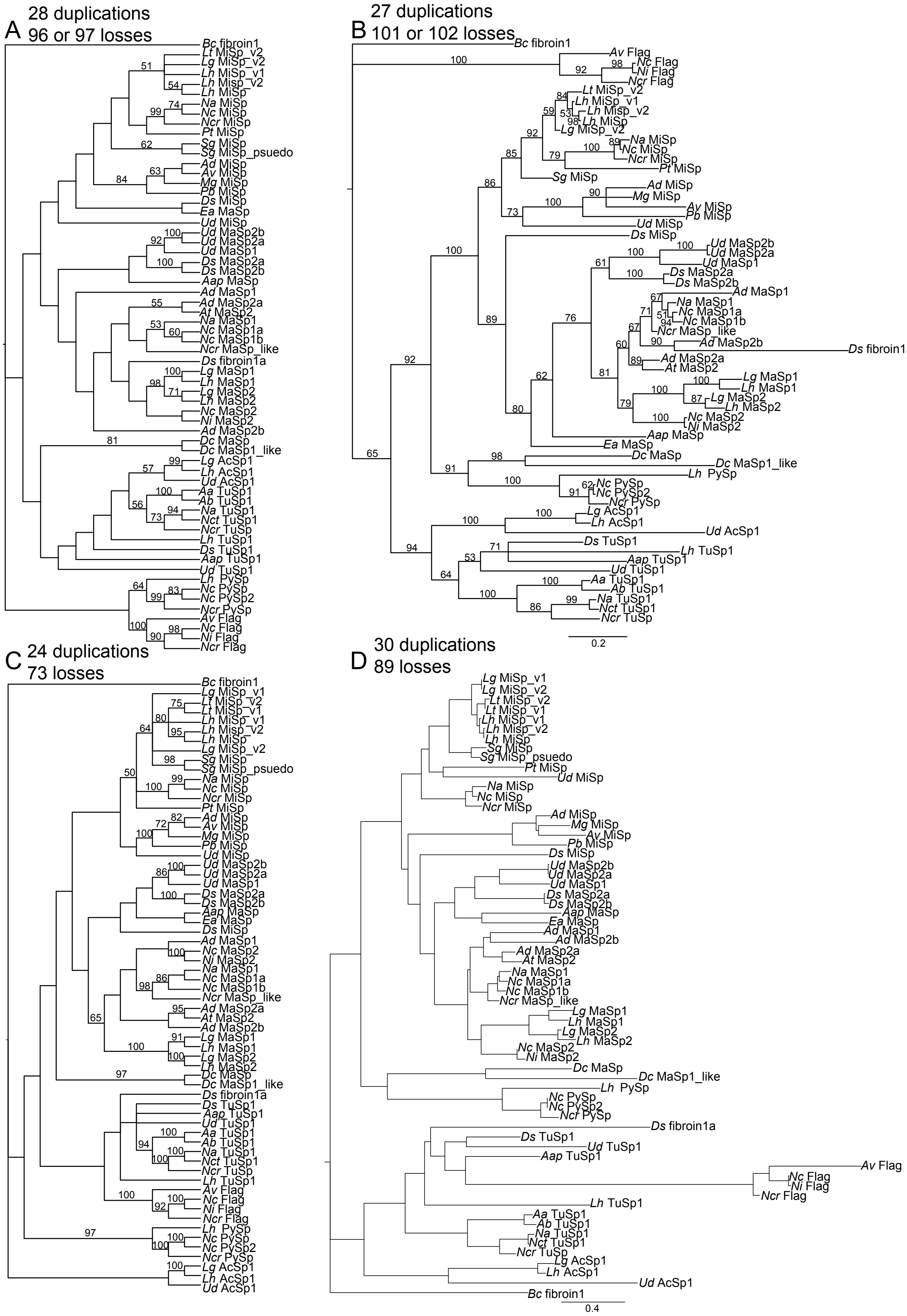
A
28 duplications
96 or 97 losses
Bc fibroin1
Lt MiSp_v2
Lg MiSp_v2
Lh MiSp_v1
Lh Misp_v2
Lh MiSp
Na MiSp
Nc MiSp
Ncr MiSp
Pt MiSp
Sg MiSp
Sg MiSp_psuedo
Ad MiSp
Av MiSp
Mg MiSp
Pb MiSp
Ds MiSp
Ea MaSp
Ud MiSp
Ud MaSp2b
Ud MaSp2a
Ud MaSp1
Ds MaSp2a
Ds MaSp2b
Aap MaSp
Ad MaSp1
Ad MaSp2a
At MaSp2
Na MaSp1
Nc MaSp1a
Nc MaSp1b
Ncr MaSp_like
Ds fibroin1a
Lg MaSp1
Lh MaSp1
Lg MaSp2
Lh MaSp2
Nc MaSp2
Ni MaSp2
Ad MaSp2b
Dc MaSp
Dc MaSp1_like
Lg AcSp1
Lh AcSp1
Ud AcSp1
Aa TuSp1
Ab TuSp1
Na TuSp1
Nct TuSp1
Ncr TuSp
Lh TuSp1
Ds TuSp1
Aap TuSp1
Ud TuSp1
Lh PySp
Nc PySp
Nc PySp2
Ncr PySp
Av Flag
Nc Flag
Ni Flag
Ncr Flag
B
27 duplications
101 or 102 losses
Bc fibroin1
Av Flag
Nc Flag
Ni Flag
Ncr Flag
Lt MiSp_v2
Lh MiSp_v1
Lh Misp_v2
Lh MiSp
Lg MiSp_v2
Na MiSp
Nc MiSp
Ncr MiSp
Pt MiSp
Sg MiSp
Ad MiSp
Mg MiSp
Av MiSp
Pb MiSp
Ud MiSp
Ds MiSp
Ud MaSp2b
Ud MaSp2a
Ud MaSp1
Ds MaSp2a
Ds MaSp2b
Ad MaSp1
Na MaSp1
Nc MaSp1a
Nc MaSp1b
Ncr MaSp_like
Ad MaSp2b
Ds fibroin1a
Ad MaSp2a
At MaSp2
Lg MaSp1
Lh MaSp1
Lg MaSp2
Lh MaSp2
Nc MaSp2
Ni MaSp2
Aap MaSp
Ea MaSp
Dc MaSp
Dc MaSp1_like
Lh PySp
Nc PySp
Nc PySp2
Ncr PySp
Lg AcSp1
Lh AcSp1
Ud AcSp1
Ds TuSp1
Lh TuSp1
Aap TuSp1
Ud TuSp1
Aa TuSp1
Ab TuSp1
Na TuSp1
Nct TuSp1
Ncr TuSp
0.2
C
24 duplications
73 losses
Bc fibroin1
Lg MiSp_v1
Lt MiSp_v2
Lt MiSp_v1
Lh MiSp_v1
Lh Misp_v2
Lh MiSp
Lg MiSp_v2
Sg MiSp
Sg MiSp_psuedo
Na MiSp
Nc MiSp
Ncr MiSp
Pt MiSp
Ad MiSp
Av MiSp
Mg MiSp
Pb MiSp
Ud MiSp
Ud MaSp2b
Ud MaSp2a
Ud MaSp1
Ds MaSp2a
Ds MaSp2b
Aap MaSp
Ea MaSp
Ds MiSp
Ad MaSp1
Nc MaSp2
Ni MaSp2
Na MaSp1
Nc MaSp1a
Nc MaSp1b
Ncr MaSp_like
Ad MaSp2a
At MaSp2
Ad MaSp2b
Lg MaSp1
Lh MaSp1
Lg MaSp2
Lh MaSp2
Dc MaSp
Dc MaSp1_like
Ds fibroin1a
Ds TuSp1
Aap TuSp1
Ud TuSp1
Aa TuSp1
Ab TuSp1
Na TuSp1
Nct TuSp1
Ncr TuSp
Lh TuSp1
Av Flag
Nc Flag
Ni Flag
Ncr Flag
Lh PySp
Nc PySp
Nc PySp2
Ncr PySp
Lg AcSp1
Lh AcSp1
Ud AcSp1
D
30 duplications
89 losses
Lg MiSp_v1
Lg MiSp_v2
Lt MiSp_v2
Lt MiSp_v1
Lh MiSp_v1
Lh Misp_v2
Lh MiSp
Sg MiSp
Sg MiSp_psuedo
Pt MiSp
Ud MiSp
Na MiSp
Nc MiSp
Ncr MiSp
Ad MiSp
Mg MiSp
Av MiSp
Pb MiSp
Ds MiSp
Ud MaSp2b
Ud MaSp2a
Ud MaSp1
Ds MaSp2a
Ds MaSp2b
Aap MaSp
Ea MaSp
Ad MaSp1
Ad MaSp2b
Ad MaSp2a
At MaSp2
Na MaSp1
Nc MaSp1a
Nc MaSp1b
Ncr MaSp_like
Lg MaSp1
Lh MaSp1
Lg MaSp2
Lh MaSp2
Nc MaSp2
Ni MaSp2
Dc MaSp
Dc MaSp1_like
Lh PySp
Nc PySp
Nc PySp2
Ncr PySp
Ds fibroin1a
Ds TuSp1
Ud TuSp1
Aap TuSp1
Av Flag
Nc Flag
Ni Flag
Ncr Flag
Lh TuSp1
Aa TuSp1
Ab TuSp1
Na TuSp1
Nct TuSp1
Ncr TuSp
Lg AcSp1
Lh AcSp1
Ud AcSp1
Bc fibroin1
0.4

Figure S8. Phylogenetic trees for C-terminal spidroin sequences (see Table 1 for accessions). Consensus of 5 maximum parsimony (MP) trees for amino acid sequence (**A)** and 29 MP trees for nucleotide sequence (**C**). Bootstrap values ≥50% are shown above branches. **B.** Bayesian tree for amino acid sequences. Posterior probabilities shown. **D.** Maximum likelihood tree for encoding nucleotides. Units are substitutions per site for B and D. *Sg* MiSp_pseudo was removed from Bayesian analyses of amino acids. *Lg* MiSp_v1 and *Lt* MiSp_v2 were removed from amino acid analyses because their C-termini were < 30aa.

Duplication and loss events were inferred for each tree by reconciling with two species trees (Figure 1). Duplications were always identical, but sometimes more losses were inferred when reconciling with the tree based on Dimitrov et al. (2016) [40], shown here as the second number.
